# Exploring Cyclopentannulation as an Effective Synthetic Tool to Design Polycyclic Aromatic Hydrocarbon AIEgens for Bioimaging

**DOI:** 10.1021/acsomega.4c05526

**Published:** 2024-08-16

**Authors:** Noorullah Baig, Suchetha Shetty, Rupa Bargakshatriya, Sumit Kumar Pramanik, Bassam Alameddine

**Affiliations:** † Department of Mathematics and Natural Sciences, Gulf University for Science and Technology, Mubarak Al-Abdullah, Hawally 32093, Kuwait; ‡ Functional Materials Group, Gulf University for Science and Technology, Mubarak Al-Abdullah, Hawally 32093, Kuwait; § CSIR-Central Salt and Marine Chemicals Research Institute, Gijubhai Badheka Marg, Bhavnagar 364002, Gujarat, India

## Abstract

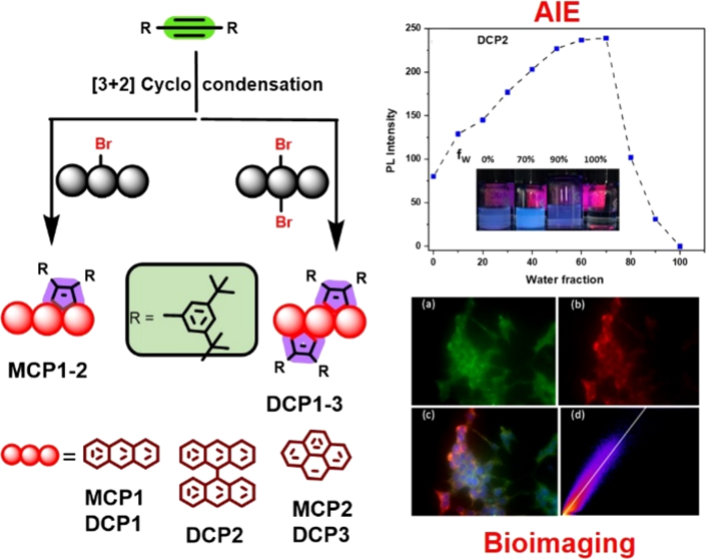

Synthesis of various polycyclic aromatic hydrocarbons (PAHs) from a palladium-catalyzed [3 + 2] cyclocondensation reaction is reported herein. The design strategy consisted of reacting the sterically hindered 1,2-bis(3,5-di*tert*-butylphenyl)acetylene **2** with myriad brominated anthracene and pyrene surrogates, resulting in the formation of target molecules **MCP1–2** and **DCP1–3**, which exhibited excellent solubility in commonly used organic solvents and unveiled prominent aggregation-induced emission (AIE) characteristics in tetrahydrofuran and water solvent mixtures. Calculations using density functional theory (DFT) were utilized to validate both the contorted structures of the target molecules and their electronic conjugation featuring HOMO–LUMO band gaps (Δ*E*) in the range of ∼2.88 to 2.97 eV for the monocylopentannulated PAHs **MCP1–2**, and between ∼2.23 to 2.41 eV for the dicyclopentannulated PAHs **DCP1–3**. Furthermore, the biomedical features of **DCP2** were investigated in cell-imaging experiments employing the RAW 264.7 macrophage cell line as a model system showing a high biocompatibility for **DCP2**, thus paving the way for its potential application in bioimaging. These findings underscore the significance of the target compounds as prominent AIEgens with exceptional photophysical properties and biocompatibility, therefore promoting them as valuable tools for bioimaging applications.

## Introduction

The ever-evolving landscape of organic chemistry has singled out polycyclic aromatic hydrocarbons (PAHs) as an exceptional family of materials, driven by their fascinating electronic properties.^1,2^ These compounds have found applications across a spectrum of cutting-edge optoelectronic technologies, encompassing organic field-effect transistors (OFETs),^3^ organic photovoltaics (OPVs),^4^ and organic light-emitting diodes (OLEDs).^5^ Among these aromatic compounds, PAHs bearing fully unsaturated pentagonal rings – commonly referred to as cyclopentafused polycyclic aromatic hydrocarbons (CP-PAHs) – have garnered significant interest.^6−8^ The latter have intrigued the researchers’ curiosity due to their unconventional properties, including a high electron affinity, notable reactivity, and promising applications in material sciences.^9−12^ The structural modifications that increase the peripheral size of cyclopentadiene- or indene-fused rings are of particular interest as they extend the π-skeletons of various conventional PAHs, such as [n]acenes, pyrene, and corannulene.^13−15^ These synthetic derivatizations induce substantial perturbations to the π-electronic system, thus impacting the overall aromatic character of the final product.

Aggregation-induced emission (AIE) is a phenomenon observed in certain molecules with intricate structural properties whereby they exhibit weak or no emission behavior in solution contrarily to an amplified luminescence upon their optimal aggregation.^8^ Interestingly, when these specially designed compounds are present as highly diluted nonaggregated molecules in solution, they undergo strong electronic-vibrational coupling, leading to substantial nonradiative transitions that quench fluorescence.^16^ Conversely, aggregation causes a molecular confinement and thus, a restriction of intramolecular motion (RIM),^17^ which weakens the electronic-vibrational coupling, reduces nonradiative transitions, and ultimately enhances the fluorescence. The AIE concept introduced by Tang et al. in 2001^18^ led to a steep surge in the quest of luminescent materials worldwide,^19,20^ given the numerous advantages AIE materials offer over traditional organic fluorophores, such as high luminescence efficiency, significant Stokes shifts, excellent biocompatibility, and good photostability.^21^ The aforementioned “turn-on” fluorescence properties promote AIE materials for their applications in chemical sensing, circularly polarized luminescence systems, biomedical imaging, disease treatment, photonic waveguides, liquid crystal displays, and OLED devices.^22−26^

In addition, the field of supramolecular chemistry, particularly in chemo- and bio-sensing, biological cell imaging, and drug delivery systems, has witnessed noticeable progress, primarily due to the advancement of fluorescent substances with high quantum yields and superior photostability.^27^ Consequently, various fluorescent materials, including pyrene, tetraphenylethyne (TPE) and naphthalenediimide (NDI) derivatives, Schiff’s bases, conjugated polymers, metal–organic frameworks (MOFs), and carbon dots (CDs), have gained interest in various applications, mainly, sensing contaminants as well as cell imaging and drug delivery.^28−31^ Recently, our group reported the synthesis of cyclopentannulated polycyclic aromatic hydrocarbon derivatives whose seemingly trivial decoration with a few t-butyl peripheral groups bestowed them with promising aggregation-induced emission properties^8^ when compared to similar PAHs with alkyl or phenyl groups.^32,33^

We report herein the synthesis of a new derivative series of cyclopentafused polycyclic aromatic hydrocarbons containing the more sterically demanding 3,5-di-*t*-butylphenyl peripheral groups, which constitute a bulky “clad” surrounding the aromatic core and affecting the aggregation behavior. Therefore, single **MCP1–2** and doubly **DCP1–3** cyclopentannulated PAHs^32^ were synthesized by reacting the sterically hindered 1,2-bis(3,5-di*tert*-butylphenyl)ethyne derivative **2** with numerous commercially available mono- and di-bromo polycondensed aromatic compounds via an optimized cyclopentannulation reaction condition.^33^ All new compounds were thoroughly characterized by using instrumental analytical techniques. These new molecules were investigated as bioimaging agents for leukemia macrophage cell lines, showing significant potential for biomedical applications. To the best of our knowledge, there have not been any previous reports on the possible use of such compounds as aggregation-induced emission enhancers (AIEgens).

## Results and Discussion

### Synthesis

**Scheme 1:**
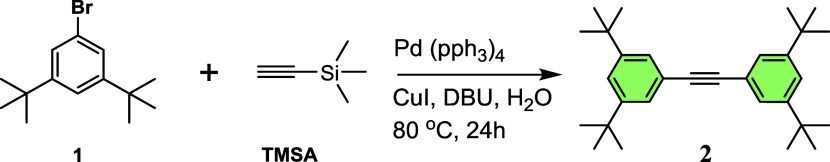
Synthesis of **2**

The synthesis of 1,2-bis(3,5-di*tert*-butylphenyl)ethyne **2** is depicted in Scheme 1, which was prepared through an archetypal Pd-catalyzed Sonogashira reaction^8,34,35^ of 1-bromo-3,5-di*tert*-butylbenzene **1** and ethynyltrimethylsilane **TMSA** resulting in the isolation of the desired compound **2** in 87% yield, which exhibited excellent dissolution in commonly used organic solvents, including DCM, chloroform, THF, and toluene. The structure of surrogate **2** was verified through various analytical techniques, including ^1^H and ^13^C nuclear magnetic resonance (NMR) spectroscopy and FT-IR spectroscopy (see Figures S1, S7, and S17).

Scheme 2 illustrates the successful mono- and two-fold Pd-catalyzed cyclopentannulation reactions^32,33^ of the highly sterically hindered tolane derivative 1,2-bis(3,5-di*tert*-butylphenyl)ethyne **2** with myriad commercially available PAHs, namely, 9-bromoanthracene **3a**, 1-bromopyrene **3b**, 9,10-dibromoanthracene **3c**, 10,10′-di-bromo-9,9′-bianthracene **3d**, and 1,6-dibromopyrene **3e**. The cyclocondensation reactions afforded the edge-extended PAH target compounds, in particular, **MCP1–2** and **DCP1–3**, in excellent yields ranging from 88 to 95%. It is noteworthy that the **MCP1–2** and **DCP1–3** exhibited excellent solubility in commonly used organic solvents and their formation was confirmed by a series of analytical techniques, such as ^1^H- and ^13^C-nuclear magnetic resonance (NMR) spectroscopy, electron impact high-resolution mass spectrometry (EI-HRMS), Fourier transform infrared (FT-IR) spectroscopy, and UV–visible absorption and fluorescence spectroscopy among others (see Figures 1–3, S2–S6, S8–S16, and S18–S26).

**Scheme 2:**
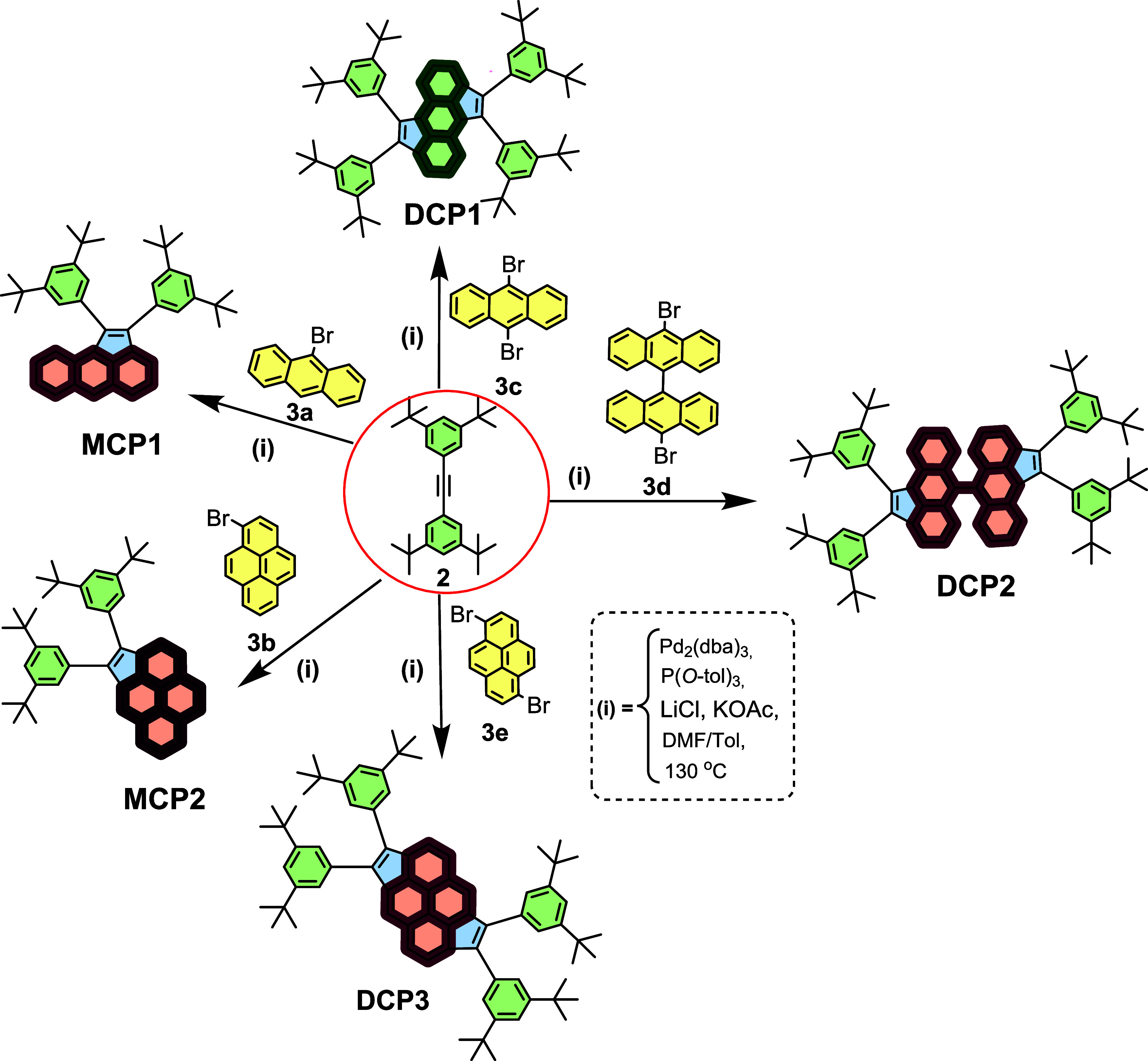
Synthesis of **MCP1–2** and **DCP1–3**

**Figure 1:**
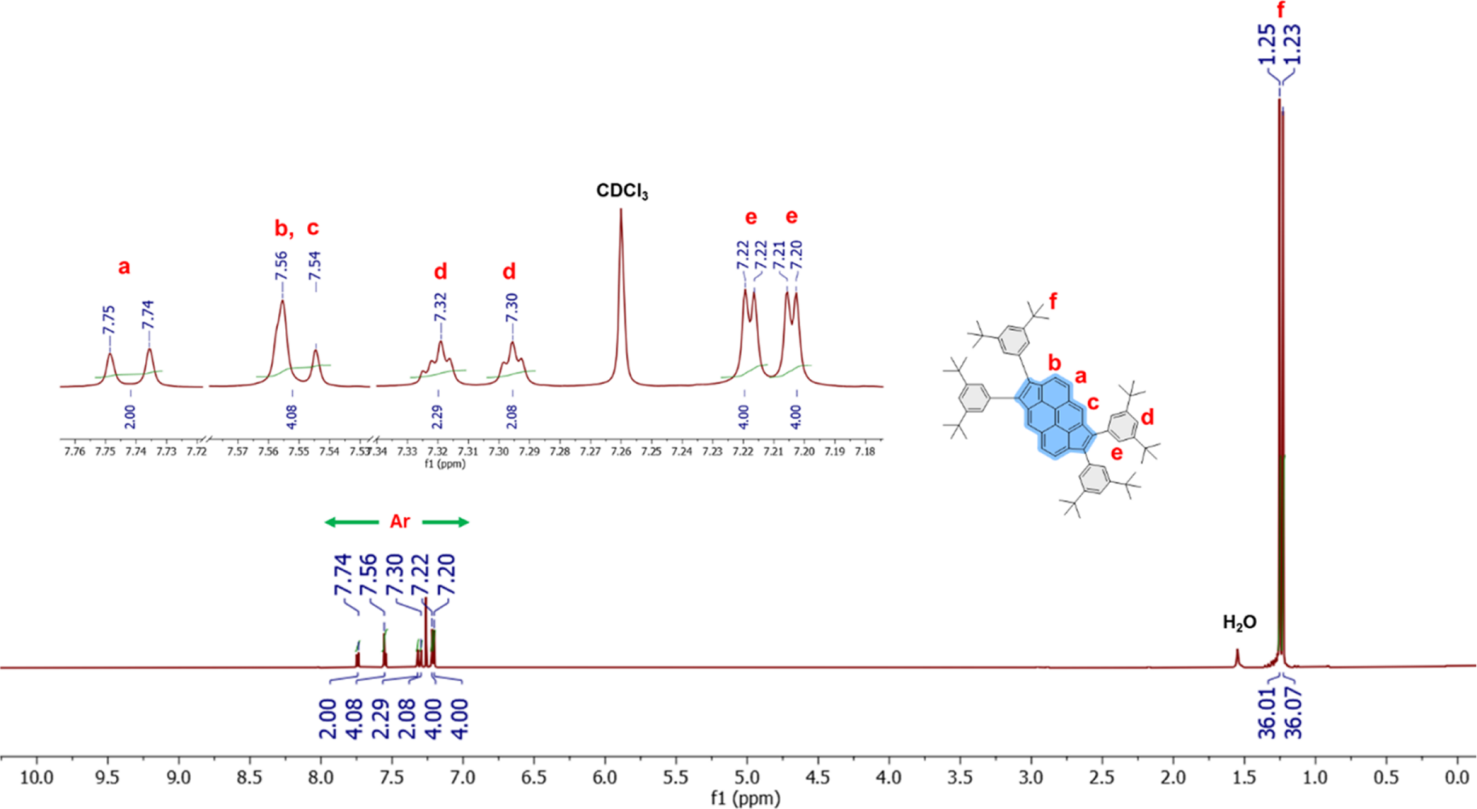
Representative ^1^H NMR of **DCP3** recorded in CDCl_3_.

Figure 1 depicts the ^1^H NMR spectra of the doubly cyclopentannulated PAH **DCP3**, showing the existence of all anticipated aromatic protons in the range of 7.7–7.2 ppm. The ^1^H NMR spectrum also exhibited the characteristic aliphatic peaks for *t*-butyl moieties at 1.25–1.23 ppm (c.f. peaks labeled f in Figure 1), whereas in the ^13^C NMR spectrum, peaks were observed at 34.8 and 31.4 ppm, corresponding to the sp^3^ carbons of the peripheral *t*-butyl groups in **DCP3** (see Figure S12). Similarly, the ^1^H- and ^13^C NMR spectra of the target compounds **MCP1–2** and **DCP1–2** display all of the distinctive chemical shifts that prove their formation (see Figures S2–S6 and S8–S12). Electron impact high-resolution mass spectrometry (EI-HRMS) corroborated the formation of **MCP1** and **DCP1–3**, elucidating their corresponding exact mass peaks (see Figures S13–S16).

**Figure 2:**
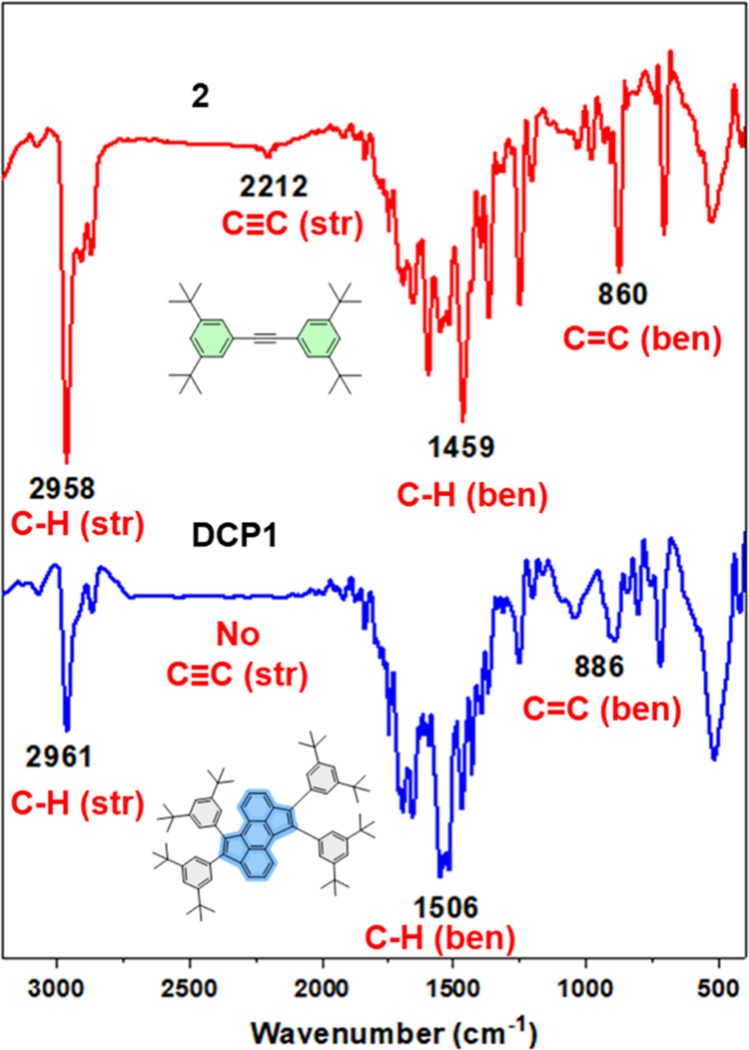
Comparative FT-IR absorption spectra of **2** (red) and the target compound **DCP1** (blue).

Figure 2 depicts the correlative FT-IR absorption spectra of synthon **2** and the desired compound **DCP1**. The distinctive stretching vibrations of the alkynyl group (CΞC) in **2** are observed at 2212 cm^–1^,^36^ which are undetected in the spectrum of **DCP1**, hence ascertaining the completion of the cyclopentannulation reaction. The FT-IR spectrum of **DCP1** conspicuously reveals the presence of aliphatic C–H stretching at 2961 cm^–1^ and bending vibrations at 1506 cm^–1^.^37^ In addition, the peaks detected at 886 cm^–1^ are ascribed to C=C bending vibrations,^37^ which further supports the successful cycloaddition reaction affording the desired PAH compound **DCP1**. Similarly, the FT-IR spectra of **MCP1–2** and **DCP1–2** PAHs display all of the typical stretching and bending vibrations, therefore further supporting their successful synthesis (see Figures S17–S22).

### Photophysical Properties

**Figure 3:**
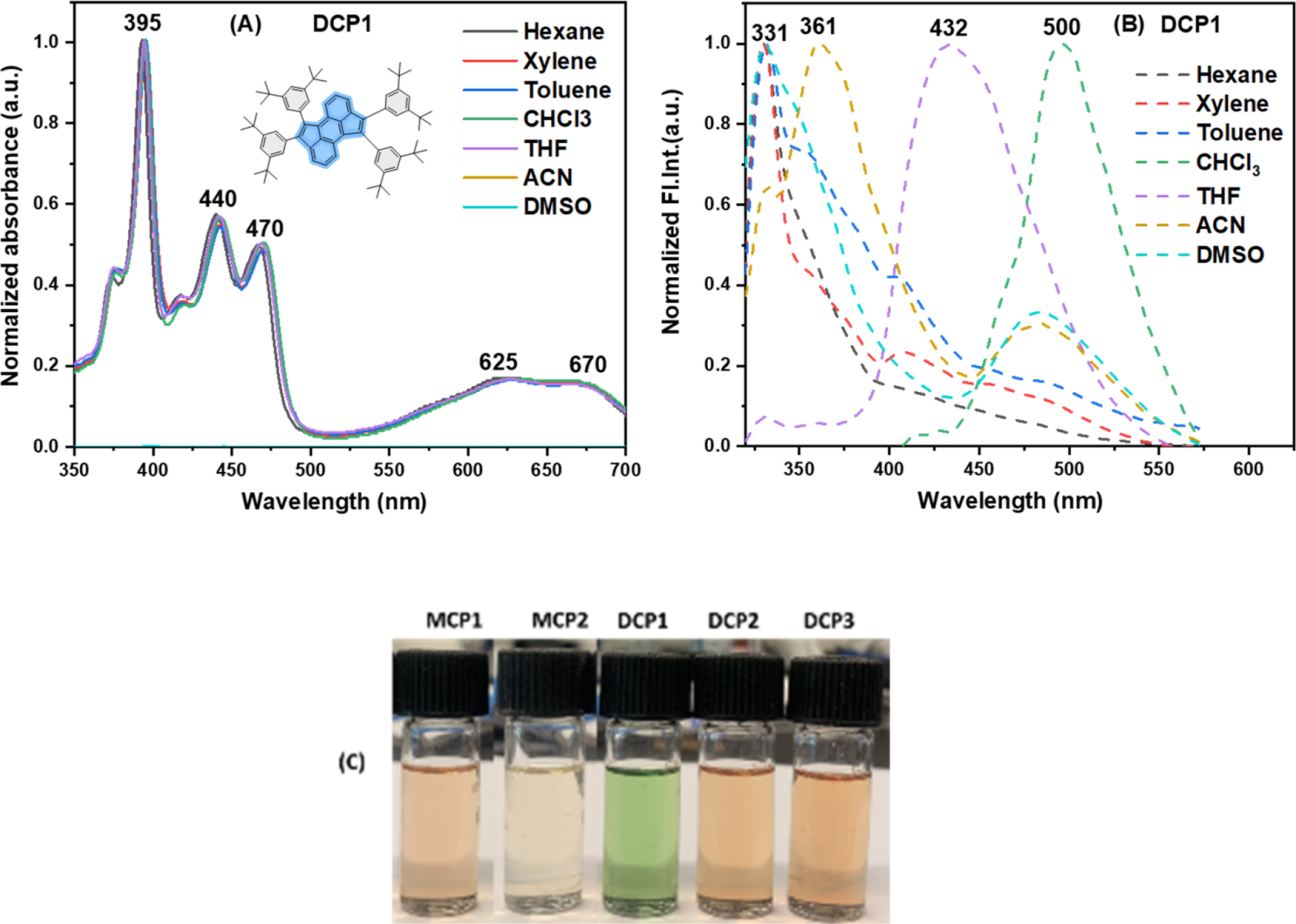
Normalized absorption (A) and emission (B) spectra of compound **DCP1** in different solvents; CM = 10^–6^ M (excitation wavelength = 395 nm). (C) Images of **MCP1–2** and **DCP1–3** in THF under daylight.

The UV–vis absorption and emission properties of **DCP1–3** and **MCP1–2** were explored using various solvents with different polarities, namely, hexane, xylene, toluene, chloroform, THF, acetonitrile (ACN), and DMSO (see Figures 3 and S23–S26). **DCP1** displayed a consistent UV–vis absorption spectrum in all of the above-mentioned solvents with absorption bands having a maximum peak at 395 nm and displaying additional bands at 440 and 470 nm, accompanied by shoulder peaks in the visible region at 625 and 670 nm. The UV–vis absorption spectra of the other doubly cyclopentannulated derivatives **DCP2** and **DCP3** portrayed analogous characteristics across all of the solvents, revealing a prominent absorption band at ∼380 nm for the former and at ∼343 nm for the latter. These observations can be attributed to the π–π* transition^38^ of the aromatic core^39^ (see Figures S24–S26). On the other hand, the UV–vis absorption spectrum of the monocylopentannulated anthracene **MCP1** disclosed an absorption peak at 372 nm with additional bands at 416 and 438 nm, along with a shoulder peak at 510 nm (see Figure S23). Similarly, the monocylopentannulated pyrene derivative **MCP2** depicted two maximum peaks in the range of 326–331 and 342–346 nm (see Figure S24).

The photoluminescence (PL) nature of **DCP1–3** and **MCP1–2** was explored using the solvents that were utilized to measure their UV–vis absorption. As illustrated in Figure 3, the emission spectra of **DCP1** exhibit similar patterns in hexane, xylene, toluene, and DMSO with the λ_max_ value detected at 331 nm, which is but red-shifted in ACN, THF, and CHCl_3_ by 30, 101, and 169 nm, respectively, thus indicating a more pronounced solvent effect in the emission when compared to UV–vis absorption spectra. Interestingly, the emission spectra of **DCP2–3** and **MCP1** reveal an emission maximum at ∼330 nm in DMSO, hexane, and THF, but these are slightly red-shifted by ∼20–40 nm in ACN whereas they display a major red shift by ∼120–145 nm in xylene and toluene (see Figures S23, S25 and S26). Conversely, the emission spectra of **MCP2** unveiled a minor red shift of 10–20 nm when transitioning from a nonpolar to a polar solvent (see Figure S24).

### Aggregation-Induced Emission (AIE) Studies

**Figure 4:**
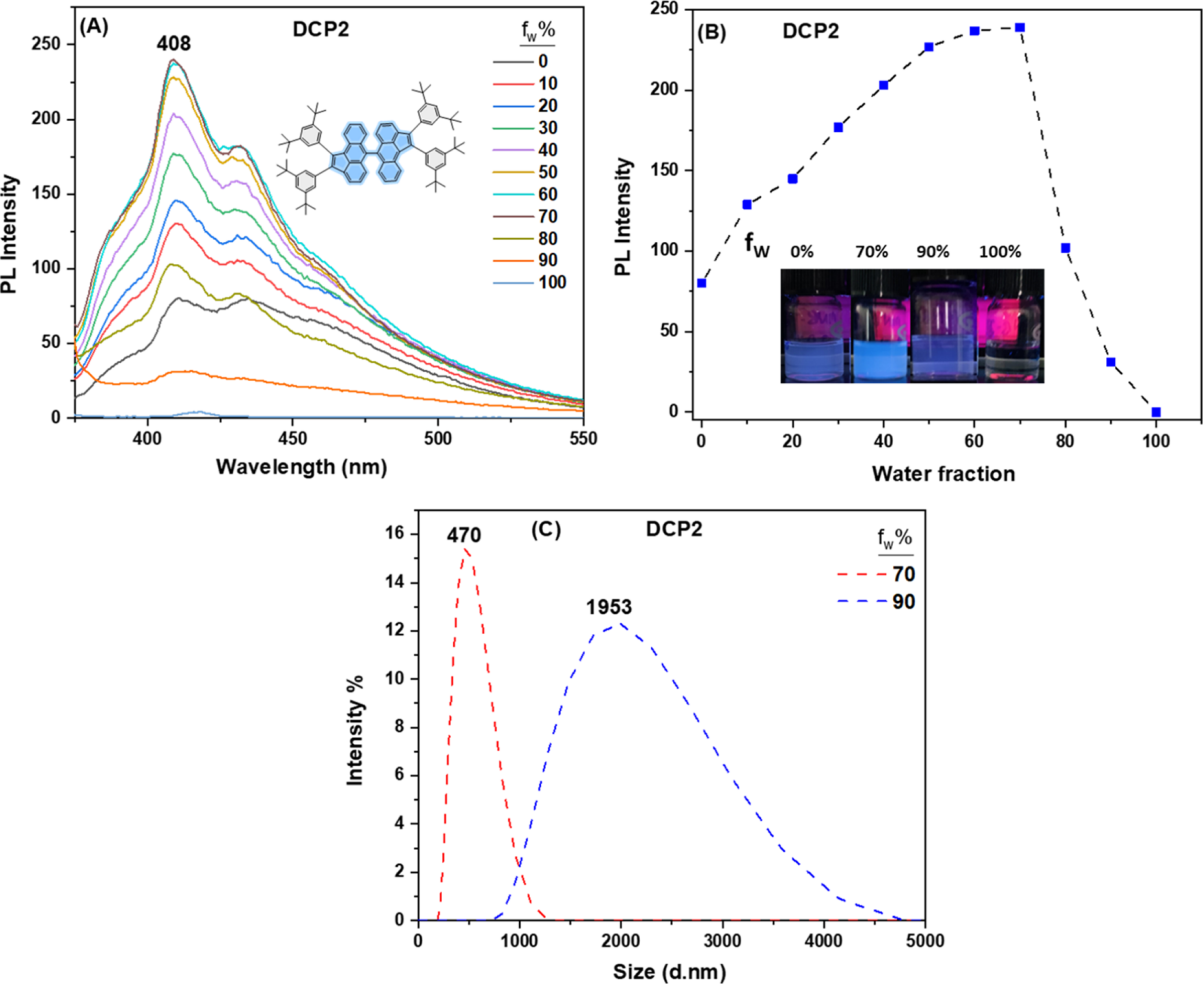
(A) Emission spectra of **DCP2** in THF/H_2_O blends (0–100%). (B) Graph depicting the maximum emission intensity of **DCP2** in relation to the water fraction. (C) Dynamic light-scattering (DLS) spectra of **DCP2** in THF/H_2_O, wt %; 70 (red dashed line) and 90 (blue dashed line).

Considering the newly synthesized PAHs **MCP1–2** and **DCP1–3** possess multiple rotation centers conducive to AIE behavior, where rotation of the peripheral phenyl rings diminishes luminescence in solution,^40^ the AIE properties of **MCP1–2** and **DCP1–3** (see Figures 4 and S27–S30) were examined in THF/H_2_O solvent blends with a rising proportion of water concentration ranging from 0 to 100%.

Figure 4 depicts the AIE investigation conducted on **DCP2**. While the solution shows a feeble emission spectrum in pure THF, the emission intensity rises upon increasing the water fraction (*f*_w_) in the medium, as could be noticed in Figure 4A,B, therefore suggesting the formation of aggregates upon the addition of water. The maximum fluorescence intensity for **DCP2** was noted at a 30:70 concentration ratio of the THF/H_2_O mixture, after which it began to decrease with the rising water fraction (*f*_w_) until it exhibited a very faint emission peak in pure water. The increase in PL intensity in the aggregated state, specifically at *f*_w_ = 70%, confirms the AIE property of **DCP2**, which is believed to arise from the interconnected rotational aromatic moieties through the conjugated spacers forming an interlocked network upon aggregation.^41^ It is worthwhile to mention that the decline in **DCP2** PL intensity when *f*_w_ > 70% can be explained by two factors: (i) the formation of large aggregates where only the molecules that are present at the surface emit light upon excitation, thus resulting in the decrease of the overall fluorescence intensity,^42^ or (ii) aggregation of the solute molecules in amorphous nanoparticle forms, leading to a reduction in PL intensity, unlike the enhancement observed with the formation of crystalline nanoparticles.^43^ However, in instances where higher *f*_w_ did not prompt precipitation, this could be explained by the formation of aggregates whose size is at the nanoscale level. Dynamic light-scattering (DLS) analysis of **DCP2** in THF/H_2_O, specifically in solutions with *f*_w_ of 70 and 90%, disclosed average aggregate sizes of approximately 470 and 1953 d.nm, respectively (Figure 4C). By correlating these findings with the change in the emission intensity observed in Figure 4B, it is evident that the reduction in emission at *f*_w_ of 90% is attributed to the fact that only the molecules at the surface of large aggregates emit light.^44^ Similarly, **MCP1–2**, **DCP1**, and **DCP3** exhibit AIE properties, each within distinct water fraction ranges. The average aggregate sizes for **MCP1** and **DCP1** fall in the range of ∼141–200 and ∼617–620 d.nm at *f*_w_ of 60 and 90%, respectively. On the other hand, **MCP2** and **DCP3** show aggregate sizes of ∼130 d.nm at an *f*_w_ of 50%, ∼ 38 d.nm at an *f*_w_ of 70%, and ∼235 and ∼214 d.nm at an *f*_w_ of 90%, respectively (see Figures S27–S30).

### Electronic Structure

**Figure 5:**
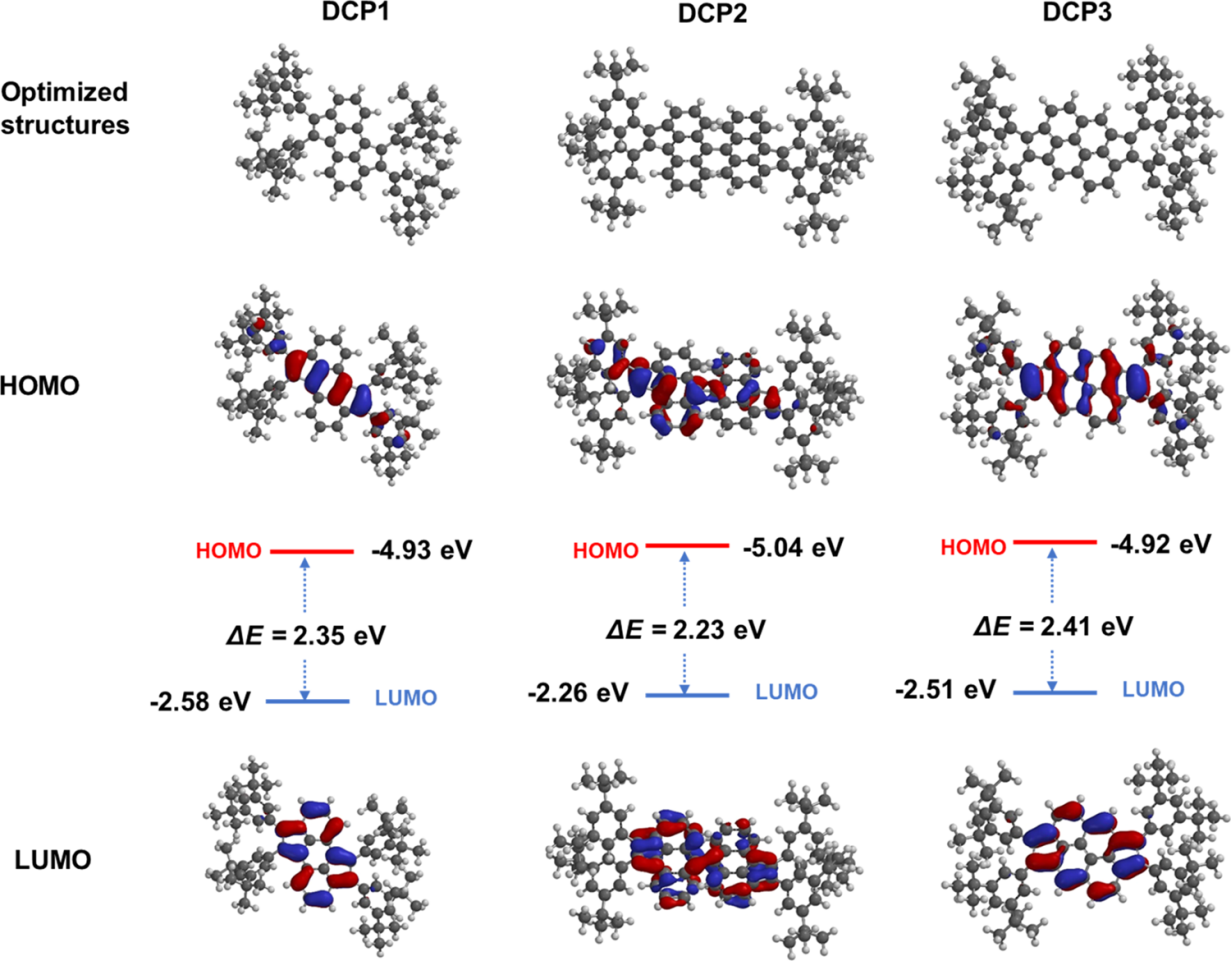
Optimized structures and molecular orbital amplitude plots of HOMO and LUMO energy levels for **DCP1–3** computed using the B3LYP/6-31G* basis set.

The optimized structures of **MCP1–2** and **BCP1–3**, employing the B3LYP/6-31G* basis set, exhibit comparable orbital distributions for the highest occupied molecular orbital (HOMO) and lowest unoccupied molecular orbital (LUMO) energy levels. Electron conjugation was found to be predominantly located within the cyclopentannulated pyrene, anthracene, and bianthracene cores. However, the conjugation is disrupted at the peripheral aryl rings due to their deviation from planarity, thus preventing their conjugation with the central aromatic core (Figure 5). It is worthwhile to mention that no dihedral angles were revealed in the resulting structures of **MCP1–2** and **DCP1–3**. The calculated HOMO and LUMO values of monocyclopentannulated PAHs **MCP1–2** are comparable (see Figure S31), revealing band gaps Δ*E* of ∼2.88–2.97 eV. On the other hand, dicyclopentannulated derivatives **DCP1–3** display lower Δ*E* values of 2.35, 2.23, and 2.41 eV, respectively.

### Cell-Imaging Studies

**Figure 6:**
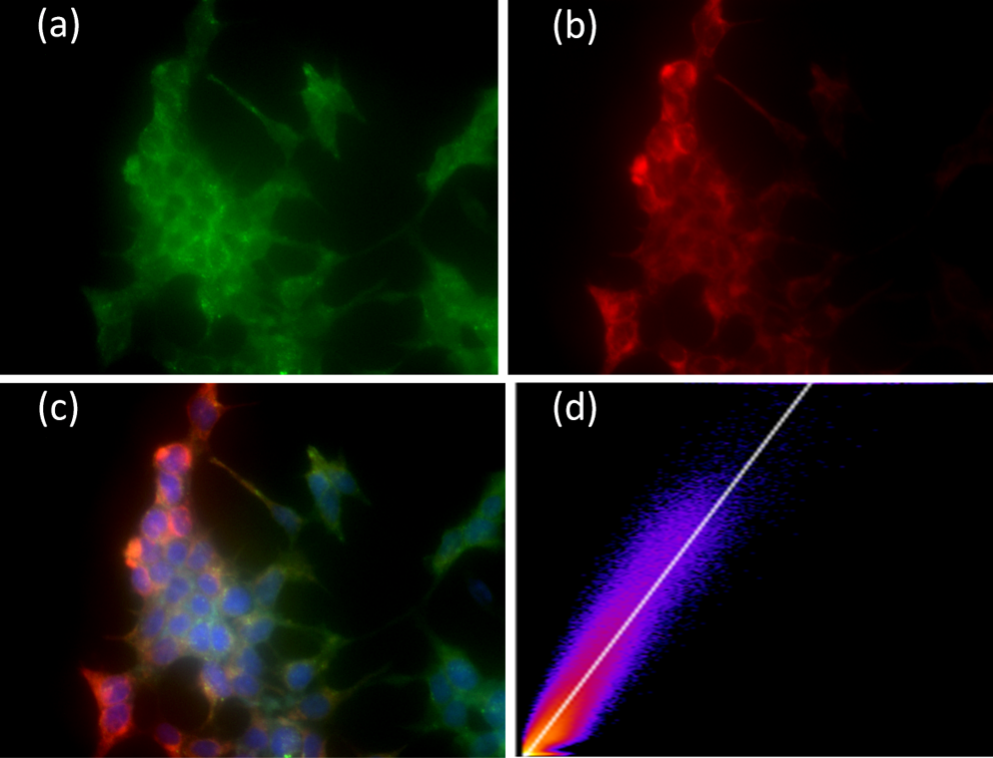
Colocalization experiments of intracellular localization of AIE dots **DCP2** using LysoTracker probes: wide-field microscopy images of cellular emission of AIE dots (a). Emission from Lyso Traker Deep Red (b). The overlap of the intensity is shown in (c). Panel (c) shows the overlap of the green and red fluorescence, indicating lysosomal localization of the UC-NPs. Panel (d) shows the Pearson coefficient = 0.82.

The cellular imaging capability of AIE dots **DCP2** was evaluated using RAW 264.7, a macrophage cell line employed as a model. Following singular incubation of the dots with RAW 264.7 cells for 6 h at a dot concentration of 5 nM, the fluorescent signal emitted by the **DCP2** was observed using emission microscopy (Figure 6). The sample was excited using a 458 nm laser, and the fluorescence signal was captured concurrently using 480–560 and 670–800 nm band-pass filters. The vivid green fluorescence observed (see Figure S32) indicates the successful labeling of the cells by **DCP2**. It is worth mentioning that the emission maxima of **DCP2** consistently fell within the 480–546 nm range in various solvents when an excitation wavelength of 458 nm was used (see Figure S33).

To investigate the potential delivery of AIE dots **DCP2** to lysosomes, LysoTracker Deep Red (LTDR) was employed, where the spectral characteristics of LTDR were found to be complementary to those of **DCP2**. The latter were one-photon excited at 488 nm, and the emission was collected at 500 to 550 nm, whereas LTDR was excited at 644 nm and emitted at 655 nm. Analysis of the intensity profile in wide-field images indicated an overlap between LysoTracker Deep Red signals and **DCP2**. The calculated Pearson’s coefficient was found to be 95%, further affirming the exclusive localization that the **DCP2** are localized in the lysosomes of RAW cells (Figure 6).

**Figure 7:**
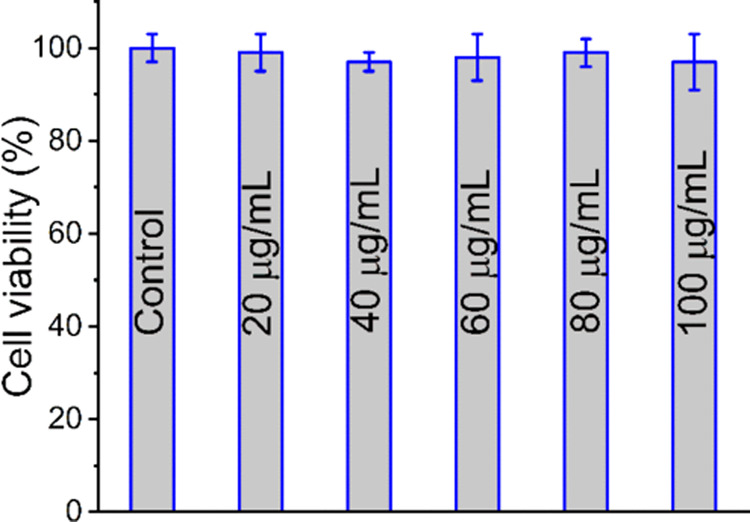
Cell viability of RAW 264.7 cells after 24 h of exposure to a concentration range of 0–100 μg/mL of **DCP2**, determined using the MTT assay. Data represent mean ± standard deviation of three replicates.

Biocompatibility studies conducted using the MTT assay on AIE dots revealed stable viability levels in treated cells compared with the control group. Notably, no decrease below 99% was reported even after exposure to various concentrations of **DCP2** for up to 24 h (Figure 7), which confirms the full biocompatibility of **DCP2**, therefore establishing them as promising candidates for biomedical applications.

## Conclusion

In conclusion, this study successfully synthesized novel polycyclic aromatic hydrocarbons (PAHs) with high efficiency by utilizing a versatile palladium-catalyzed cyclopentannulation reaction. Through a thorough examination of the optical properties of the desired compounds, namely **MCP1–2** and **DCP1–3**, we uncovered their fascinating behavior known as aggregation-induced emission (AIE) when placed in solvent mixtures of THF and H_2_O. Structural optimization studies carried out by density functional theory (DFT) calculations at the B3LYP/6-31G* basis set unveiled that the electronic conjugation is located within the core structures of the cyclopentannulated PAHs, but is disrupted at the peripheral aryl rings due to their deviation from planarity, thus preventing their effective conjugation with the aromatic center.

Furthermore, our bioimaging investigations using the RAW 264.7 macrophage cell line as a model system unequivocally demonstrate the full biocompatibility of **DCP2**. This research output not only advances our comprehension of these innovative PAHs’ distinctive photophysical traits but also opens up exciting possibilities for their utilization across a diverse spectrum of scientific and technological domains, particularly within the domains of optoelectronics and biomedicine.

## Data Availability

The raw data required to reproduce these findings are available upon request.

## Supporting Information Available

Experimental procedure, characterization of all compounds, and copies of NMR, EI-HRMS, FT-IR, UV–vis absorption and Emission spectra and AIE studies (PDF)
