# Supplementary material for: Exploring Cyclopentannulation as an Effective Synthetic Tool to Design Polycyclic Aromatic Hydrocarbon AIEgens for Bioimaging
Source: ACS Omega. 2024 Aug 16;9(34):36732–40. doi: 10.1021/acsomega.4c05526 (PMC11360036; doi:10.1021/acsomega.4c05526)
Supplement: Supplementary file 1 — ao4c05526_si_001.pdf [file ao4c05526_si_001.pdf]

Supplementary Information

Exploring Cyclopentannulation as an Effective Synthetic Tool to Design Polycyclic Aromatic Hydrocarbon AIEgens for Bioimaging

Noorullah Baig[a,b], Suchetha Shetty[a,b], Rupa Bargakshatriya[c], Sumit Kumar Pramanik[*c], Bassam Alameddine[*a,b]

[a] Department of Mathematics and Natural Sciences, Gulf University for Science and Technology, Mubarak Al-Abdullah, Hawally 32093, Kuwait.

[b] Functional Materials Group, Gulf University for Science and Technology, Mubarak Al-Abdullah, Hawally 32093, Kuwait.

[c] CSIR-Central Salt and Marine Chemicals Research Institute, Gijubhai Badheka Marg, Bhavnagar, Gujarat 364002, India.

*†Correspondence: *E-mail address*: alameddine.b@gust.edu.kw; Tel: +965 2530 7111

Contents

Experimental section	
^{1}H NMR spectra of **2, MCP1-2**, and **DCP1-3**	Figure S1-S6
^{13}C NMR spectra of **2, MCP1-2,** and **DCP1-3**	Figure S7-S12
EI-HRMS spectra of **MCP1, and DCP1-3**	Figure S13-S16
FT-IR spectra of **2, MCP1-2** and **DCP1-3**	Figure S17-S22
UV-Vis absorption and Emission spectra of **MCP1-2** and **DCP2-3**	Figure S23-S26
AIE studies of **MCP1-2, DCP1** and **DCP3** in THF/water mixture	Figure S27-S30
Optimized structures of **MCP1** and **MCP2**	Figure S31
Optical microscopy images of **DCP2** and RAW cells	Figure S32
Emission spectra of **DCP2** in different solvents excited at 458nm	Figure S33

EXPERIMENTAL SECTION

General Information

All reactions were conducted under an inert atmosphere of dry argon. Chemical reagents were obtained from Merck (Darmstadt, Germany) and HiMedia (Mumbai, India) and were employed as purchased without any further purification, except where noted otherwise. Iscove's Modified Dulbecco's Medium (IMDM), Dulbecco's Modified Eagle's medium (DMEM), fetal calf serum (FCS), phosphate buffer saline (PBS), fetal bovine serum (FBS), penicillin–streptomycin, 4% paraformaldehyde (PFA), Vectashield h-1000 (mounting agent), 50 mM ammonium chloride, Lyso tracker green, Lyso tracker deep red, and Hoechst (33342) were purchased from Sigma-Aldrich and were used without any further purification. Penicillin/streptomycin (P/S) rhodamine green, and sodium dodecyl sulfate (SDS) were obtained from Fischer scientific and were used as received. The synthesis of 1,2-bis(3,5-di-tert-butylphenyl)ethyne (**2**) followed previously established procedures.[29] [35] Solvents such as hexane, chloroform ($CHCl_3$), dichloromethane (DCM), tetrahydrofuran (THF), xylene, toluene, methanol, diisopropylamine, acetonitrile (ACN), dimethylsulfoxide (DMSO), and dimethylformamide (DMF) were dried over molecular sieves and deoxygenated by purging with argon gas for 30 minutes prior to use. Thin-layer chromatography (TLC) was performed on aluminum sheets coated with silica gel 60 F254 and visualized under a UV lamp. Nuclear Magnetic Resonance spectra, including ^1H NMR (600 MHz) and ^{13}C NMR (150 MHz), were recorded using a JEOL resonance ECZ600R spectrometer at 25°C. $CDCl_3$ served as the solvent, and chemical shifts (δ) were reported in parts per million (ppm), referenced to tetramethylsilane (TMS). Electron impact high-resolution mass spectra (EI-HRMS) were acquired on a Thermo (DFS) instrument, utilizing standard perfluorokerosene (PFK) as a lock mass. The data was processed into accurate mass values using X-Calibur accurate mass calculation software. UV-Vis spectra were recorded on a Shimadzu UV1800 spectrophotometer. Photoluminescence (PL) spectra were obtained using an Agilent G9800 Cary Eclipse Fluorescence spectrophotometer. Fourier Transform Infrared Spectroscopy (FT-

IR) spectra were recorded on a PerkinElmer G spectrophotometer with a KBr matrix. Both size and size distribution of the dye molecule and their aggregates were measured at 25°C by dynamic light-scattering (DLS) using a Brookhaven instruments Zetapals (USA) and expressed as diameter values in nanometers (d.nm)**Synthetic procedure**

1,2-bis(3,5-di-tert-butylphenyl)ethyne ***2***

A Schlenk tube was charged under an inert atmosphere of argon with 1-bromo-3,5-di-tert-butylbenzene **1** (2.0 g, 7.5 mmol, 1 eq.), ethynyltrimethylsilane **TMSA** (0.520 mL, 3.75 mmol, 0.5 eq.), tetrakis(triphenylphosphine)palladium (0) [$Pd(PPh_3)_4$] (520 mg, 0.45 mmol, 6 mol%), 1,8-diazabicyclo[5.4.0]undec-7-ene (DBU, 6.7 mL, 45 mmol), and CuI (142 mg, 0.75 mmol) in 10 mL of a deoxygenated toluene/water mixture (10 : 0.054 v/v). The reaction mixture was refluxed overnight, and the solvent was then evaporated under reduced pressure. The resulting residue was extracted with DCM from an aqueous solution of 10% HCl (80 mL) and the organic layer was washed with a brine solution followed by deionized water (100 mL × 2). The desired product was isolated by silica gel column chromatography, using DCM/hexane (10 : 90 v/v) as the eluent. Pale yellow solid (1.3 g, 87%). ^{1}H NMR (600 MHz, $CDCl_3$, ppm): δ 7.41 (d, 4H, J = 1.7 Hz,), 7.39 (t, 2H, J = 1.9 Hz,), 1.34 (s, 36H,). ^{13}C NMR (150 MHz, $CDCl_3$, ppm): δ 150.8, 125.9, 122.6, 122.5, 89.3, 34.9, 31.4. FTIR (KBr, cm^{-1}): 2958 (C-H str), 2212 (C≡C str), 1459 (C-H ben), 860 (C=C ben).

1,2-bis(3,5-di-tert-butylphenyl)aceanthrylene ***MCP1*** *(procedure B)*

A Schlenk tube was charged under argon with 9-bromoanthracene **3a** (98 mg, 0.38 mmol, 1 eq.), **2** (153 mg, 0.38 mmol, 1 eq.), Tris(dibenzylideneacetone)dipalladium(0) [$Pd_2(dba)_3$] (34 mg, 0.038 mmol, 0.1 eq), tris(o-tolyl)phosphine (P(*o*-tol)$_3$, (11.5 mg, 0.038 mmol), Potassium acetate, (KOAc) (34 mg, 0.35 mmol), Lithium chloride (LiCl) (6.0 mg, 0.14 mmol), DMF/toluene (5 mL, 1:1 v/v) and the solution was refluxed overnight. The solvent was evaporated under reduced pressure and the resulting mixture was dissolved in DCM and extracted with a saturated solution of $NaHCO_3$ (25 mL x 2). The combined organic layer was

washed with deionized water (50 mL × 3), concentrated, and the product was purified by silica gel column chromatography using DCM/hexane (20:80 v/v) as eluent Affording a red solid (200 mg, 91%). ^{1}H-NMR (600 MHz, $CDCl_3$, ppm): δ 8.51 (*s*, 1H,), 8.10 (*t*, 2H, J = 9.7 Hz,), 8.04 (*d*, 1H, J = 8.2 Hz,) 7.88 (d, 1H, J = 6.6 Hz,), 7.65 (t, 1H, J = 8.2 Hz,), 7.38 (br, 1H,), 7.36 (m, 1H,), 7.33 (d, 2H, J = 1.7 Hz,), 7.30 (m, 1H,), 7.25 (t, 1H, J = 2.0 Hz,), 7.18 (*d*, 2H, J = 2.0 Hz,), 1.25 (*s*, 18H,), 1.21 (*s*, 18H,). ^{13}C-NMR (150 MHz, $CDCl_3$, ppm): δ 150.3, 150.1, 141.9, 139.9, 139.0, 136.8, 135.0, 134.5, 130.6, 130.2, 128.6, 128.0, 127.3, 126.9, 126.5, 125.5, 124.9, 124.8, 124.4, 120.3, 120.1, 34.8, 31.5, EI-HRMS: m/z calculated for $M^{\bullet+}$ $C_{44}H_{50}$ 578.3913 found 578.3912. FTIR (KBr, cm^{-1}): 2961 (C-H str), 1466 (C-H ben), 860 (C=C ben).

Synthesis of 3,4-bis(3,5-di-tert-butylphenyl)cyclopenta[cd]pyrene ***MCP2***

MCP2 was prepared following procedure B with: 1-bromopyrene **3b** (70 mg, 0.25 mmol, 1 eq.), **2** (100 mg, 0.25 mmol, 1 eq.), $Pd_2(dba)_3$ (23 mg, 0.025 mmol, 0.1 eq.), P(*o*-tol)$_3$ (7.6 mg, 0.025 mmol), KOAc (122 mg, 1.25 mmol) and LiCl (21 mg, 0.5 mmol). red solid (133 mg, 89%). ^{1}H-NMR (600 MHz, $CDCl_3$, ppm): δ 8.42 (d, 2H, *J* = 9.4 Hz,), 8.30 (d, 1H, *J* = 7.4 Hz,), 8.19 (s, 2H,), 8.13 (d, 1H, *J* = 8.8 Hz,), 8.0 (t, 2H, *J* = 8.9 Hz,), 7.39 (d, 2H, *J* = 1.9 Hz,), 7.37 (m, 4H,), 1.29 (*s*, 18H,), 1.27 (*s*, 18H,), ^{13}C-NMR (150 MHz, $CDCl_3$, ppm): δ 150.6, 143.6, 139.4, 134.9, 130.2, 130.1, 128.2, 127.2, 126.9, 126.7, 126.4, 125.7, 124.7, 124.4, 122.3, 122.0, 121.0, 120.7, 34.8, 31.5; FTIR (KBr, cm^{-1}): 2958 (C-H str), 1532 (C-H ben), 846 (C=C ben).

1,2,6,7-tetrakis(3,5-di-tert-butylphenyl)cyclopenta[hi]aceanthrylene ***DCP1***

DCP1 was prepared following procedure B with: 9,10-dibromoanthracene **3c** (64 mg, 0.19 mmol, 1 eq.), **2** (153 mg, 0.38 mmol, 2 eq.), $Pd_2(dba)_3$ (18 mg, 0.019 mmol, 0.1 eq.), P(o-tol)$_3$ (6.0 mg, 0.019 mmol), KOAc (93 mg, 0.95 mmol) and LiCl (16 mg, 0.38 mmol). green solid (175 mg, 93%). ^{1}H-NMR (600 MHz, $CDCl_3$, ppm): δ 7.98 (d, 2H, J = 8.5 Hz,), 7.78 (d, 2H, J = 6.3 Hz,), 7.44 (t, 2H, J = 6.9 Hz,), 7.38 (s, 6H,), 7.26 (m, 2H,), 7.20 (d, 4H, J = 1.6 Hz,), 1.27 (s, 36H,), 1.22 (s, 36H,), ^{13}C-NMR (150 MHz, $CDCl_3$, ppm): δ 150.3, 140.8, 139.8, 137.8,

136.3, 134.3, 128.1, 126.4, 125.5, 125.3, 124.8, 120.5, 120.3, 34.8, 31.5; EI-HRMS: m/z calculated for $M^{•+}$ $C_{74}H_{90}$ 978.7043 found 978.7061. FTIR (KBr, cm^{-1}): 2961 (C-H str), 1506 (C-H ben), 886 (C=C ben).

1,1',2,2'-Tetrakis(3,5-di-tert-butylphenyl)-6,6'- biaceanthrylene ***DCP2***

DCP2 was prepared following procedure B with: 10,10′-dibromo-9,9′-bianthracene **3d** (97 mg, 0.19 mmol, 1 eq.), **2** (153 mg, 0.38 mmol, 2 eq.), $Pd_2(dba)_3$ (18 mg, 0.019 mmol, 0.1 eq.), $P(o\text{-}tol)_3$ (6.0 mg, 0.019 mmol), KOAc (93 mg, 0.95 mmol) and LiCl (16 mg, 0.38 mmol). Red solid (193 mg, 88%). ^{1}H-NMR (600 MHz, $CDCl_3$, ppm): δ 8.71 (d, 2H, J = 8.6 Hz,), 8.28 (d, 2H, J = 9.1 Hz,), 7.86 (d, 2H, J = 6.3 Hz,), 7.60-7.58 (m, 2H,), 7.46-7.43 (m, 6H,), 7.41 (m, 4H,), 7.29-7.28 (m, 6H,), 7.25 (m, 2H,), 1.30 (s, 36H,), 1.24 (s, 36H,), ^{13}C-NMR (150 MHz, $CDCl_3$, ppm): δ 150.5, 141.9, 136.8, 136.1, 135.0, 134.5, 132.9, 130.1, 127.9, 127.6, 127.2, 126.7, 126.1, 125.7, 125.1, 124.9, 120.4, 34.9, 31.5; EI-HRMS: m/z calculated for $M^{•+}$ $C_{88}H_{98}$ 1154.7669 found 1154.7670. FTIR (KBr, cm^{-1}): 2961 (C-H str), 1514 (C-H ben), 908 (C=C ben).

1,2,6,7-tetrakis(3,5-di-tert-butylphenyl)dicyclopenta[cd,jk]pyrene ***DCP3***

DCP3 was prepared following procedure B with: 1,6-dibromopyrene **3e** (68 mg, 0.19 mmol, 1 eq.), **2** (153 mg, 0.38 mmol, 2 eq.), $Pd_2(dba)_3$ (18 mg, 0.019 mmol, 0.1 eq.), $P(o\text{-}tol)_3$ (6.0 mg, 0.019 mmol), KOAc (93 mg, 0.95 mmol) and LiCl (16 mg, 0.38 mmol). Red solid (180 mg, 95%). ^{1}H-NMR (600 MHz, $CDCl_3$, ppm): δ 7.74 (d, 2H, J = 7.7 Hz,), 7.56 (d, 4H, J = 6.6 Hz,), 7.32 (t, 2H, J = 2.0 Hz,), 7.30 (t, 2H, J = 1.9 Hz,), 7.22 (d, 4H, J = 1.7 Hz,), 7.20 (d, 4H, J = 1.7 Hz,), 1.25 (s, 36H,), 1.23 (s, 36H,), ^{13}C-NMR (150 MHz, $CDCl_3$, ppm): δ 150.6, 141.6, 140.9, 139.8, 134.5, 132.1, 130.7, 129.5, 125.2, 124.0, 123.5, 121.7, 121.1, 34.8, 31.4; EI-HRMS: m/z calculated for $M^{•+}$ $C_{76}H_{90}$ 1002.7043 found 1002.7045. FTIR (KBr, cm^{-1}): 2958 (C-H str), 1517 (C-H ben), 886 (C=C ben).

Optical Microscopy

Macrophage RAW 264.7 cells were seeded on coverslips (22 mm × 32 mm, 180 ± 5 μm cover glasses) placed in twenty-four-well plates in DMEM cell culture medium containing 12% FBS and 2% penicillin–streptomycin for 24 h at 37 °C and 5% carbon dioxide. After 24 h when around 75% confluency was reached, these cells were washed three times with DMEM culture medium. subsequently, the cells were washed again thrice with PBS buffer. After live cell uptake for 6 h, the cells were washed again with DMEM media and fixed with 4% PFA for 20 min and then washed several times with PBS buffer, and the coverslips were mounted using the mounting medium (Vectashield h-1000). All the microscopic studies were carried out using a Delta Vision OMX-SIM microscope running in deconvoluted wide-field mode. Image processing was carried out by using the Soft Worx software.

Cellular Uptake and Imaging of AIE dots

Macrophage RAW 264.7 cells were grown in 12% FBS in DMEM with 2% penicillin/streptomycin. The confluent cells were treated with 5 nM of AIE dots for 6 h at 37 °C and 5% carbon dioxide. Cells were treated with 500 nM Lyso tracker Red for 30 min before 12 h were over. Then the cells were washed for three times with 1× PBS buffer to remove the surface adsorbed molecules. Cells were fixed using cool methanol for 120 min at −20 °C and then rehydrated for 20 min with PBS 1× buffer at 37 °C. After washing the cells with PBS 1× twice, the coverslips were mounted on glass-slides using the mounting medium. Fluorescence was observed in Zeiss microscope.

MTT Assay

The AIE dot solution was tested for cytotoxicity by MTT assay. Macrophage RAW 264.7 cells (6×10^3) were seeded in a 96-well-plate in DMEM medium supplemented with 6% FBS along with 200 units of penicillin–streptomycin antibiotics. The RAW 264.7 cells were treated with 0 to 100 μg/mL of AIE dots and incubated at 37 °C in a 5% carbon dioxide flow incubator. After

incubation for 24 h, AIE dots treated cells were washed several times with 1×PBS. The concentration range used for the current study was based on a literature value that was typically used for cell studies using AIE dots. MTT reagent (0.5 mg/mL) was added to the cells and incubated for 4 h more at 37 °C. The cell culture media was then decanting off and the formazan crystals formed were dissolved in DMSO. The percentage of cell death was calculated by measuring the absorbance reading of the formazan at 570 nm. The experiment was done in triplicate.

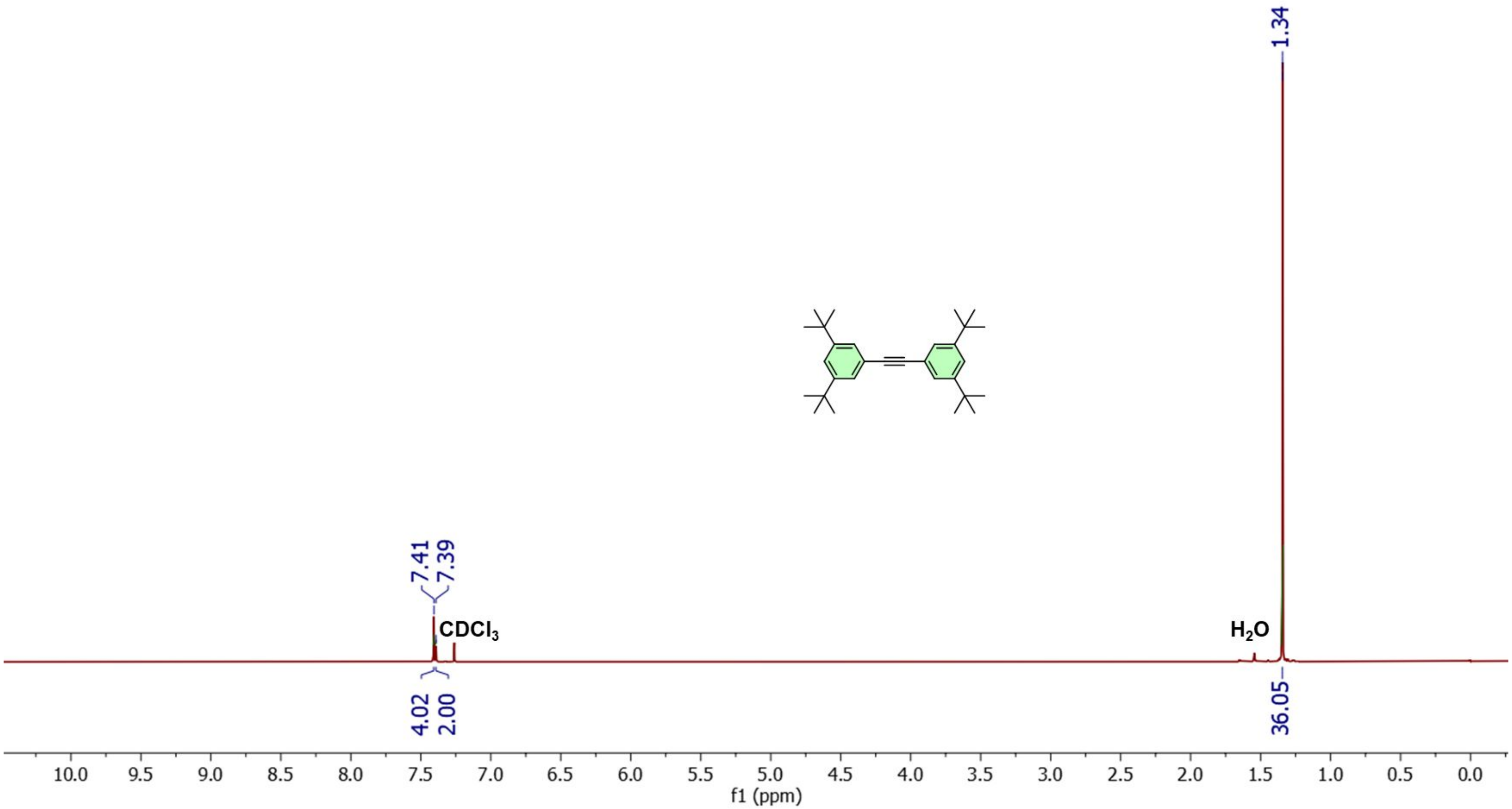

Figure S1: ^{1}H NMR spectrum of **2** ($CDCl_3$, 600 MHz)

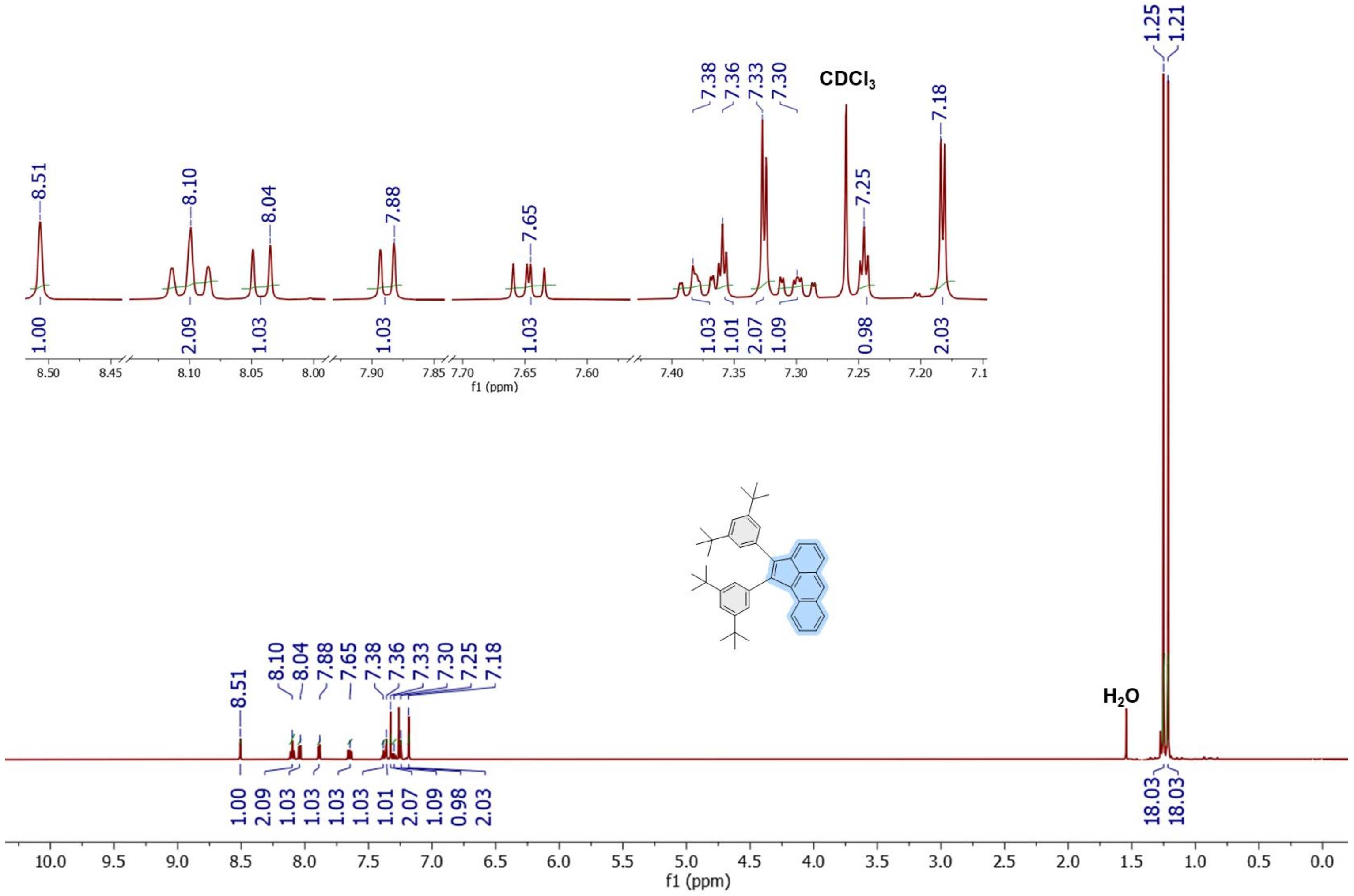

Figure S2: ^1H NMR spectrum of **MCP1** ($CDCl_3$, 600 MHz)

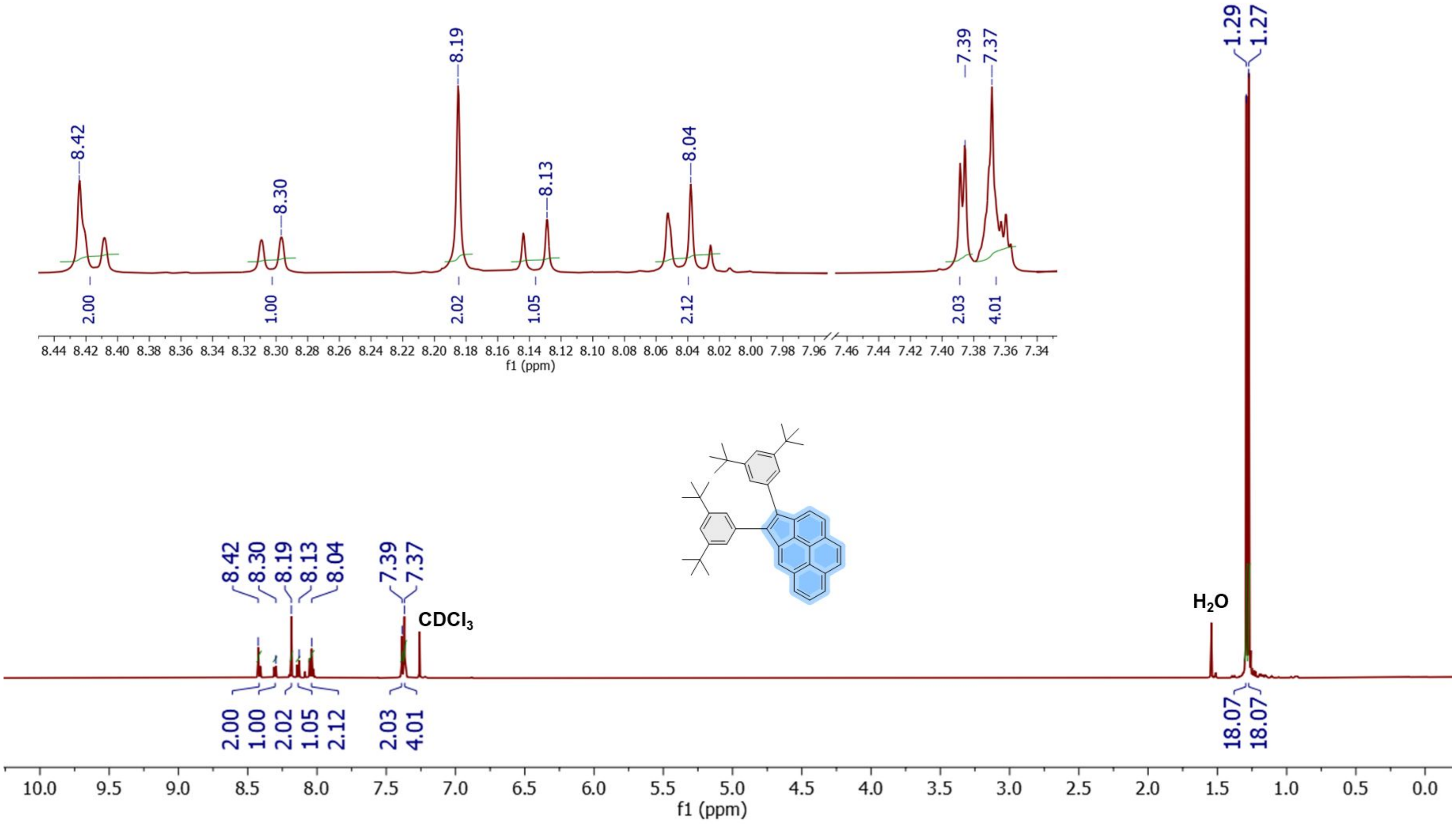

Figure S3: ^{1}H NMR spectrum of **MCP2** ($CDCl_3$, 600 MHz)

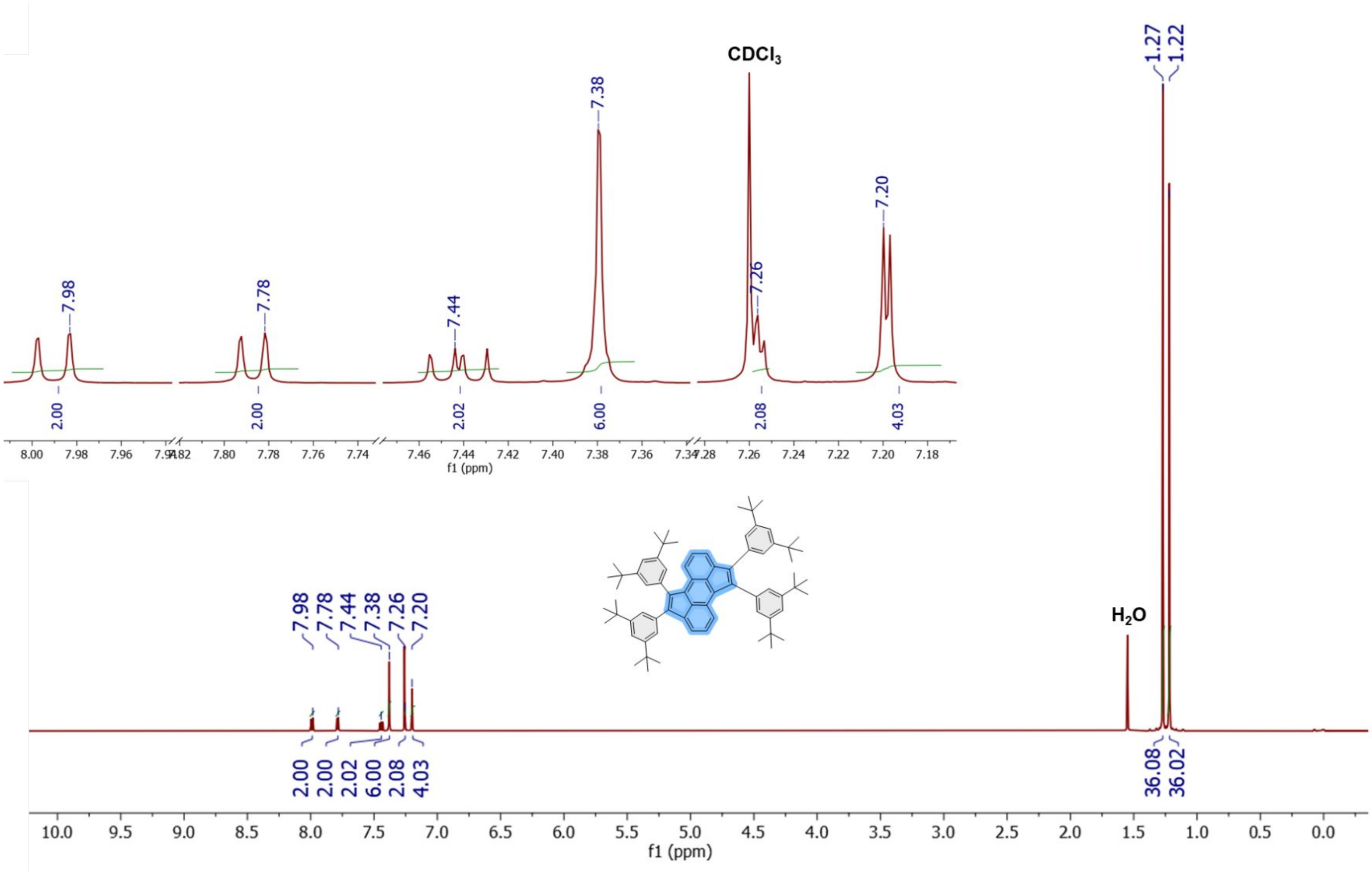

Figure S4: ^{1}H NMR spectrum of **DCP1** ($CDCl_3$, 600 MHz)

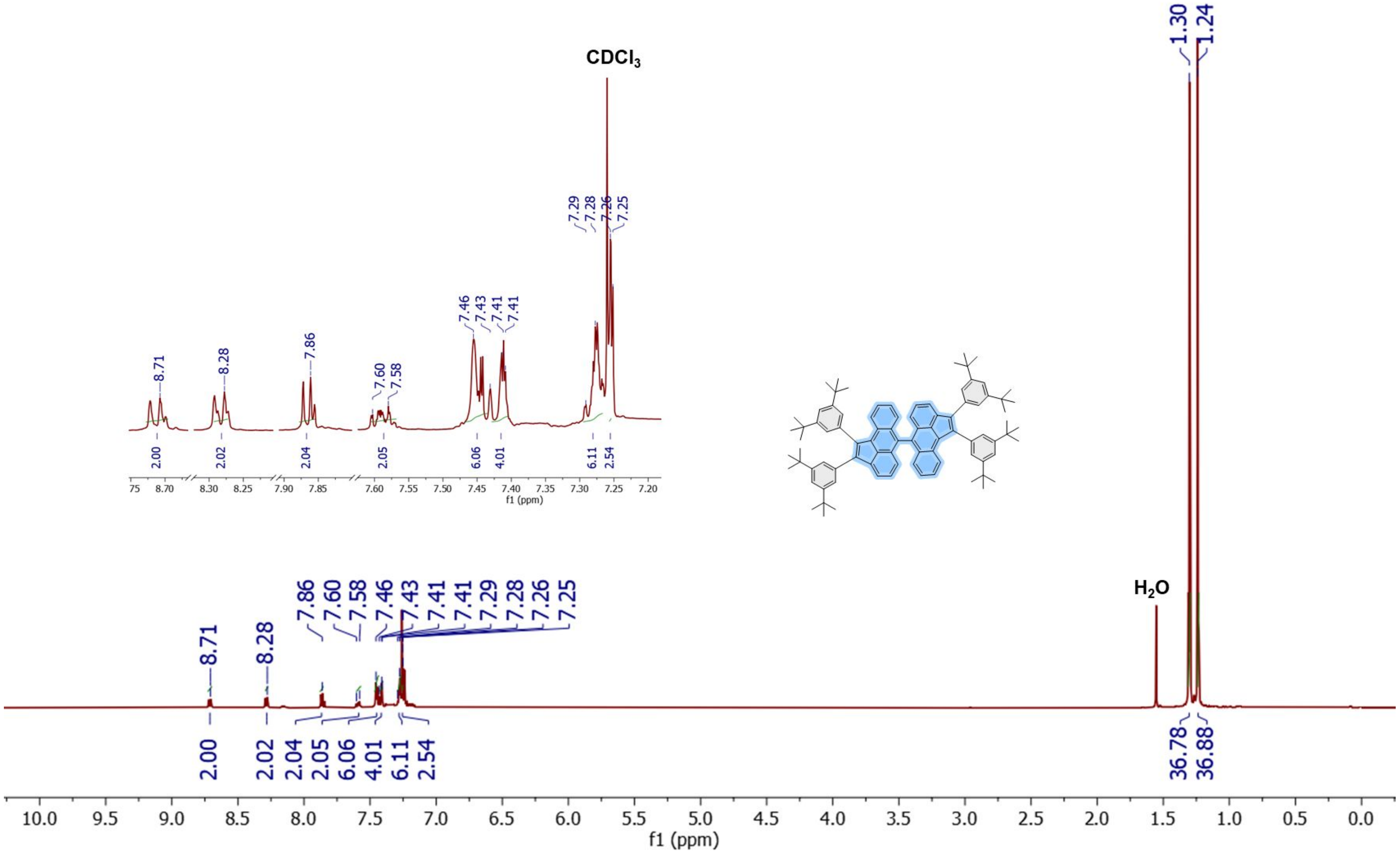

Figure S5: ^{1}H NMR spectrum of **DCP2** ($CDCl_3$, 600 MHz)

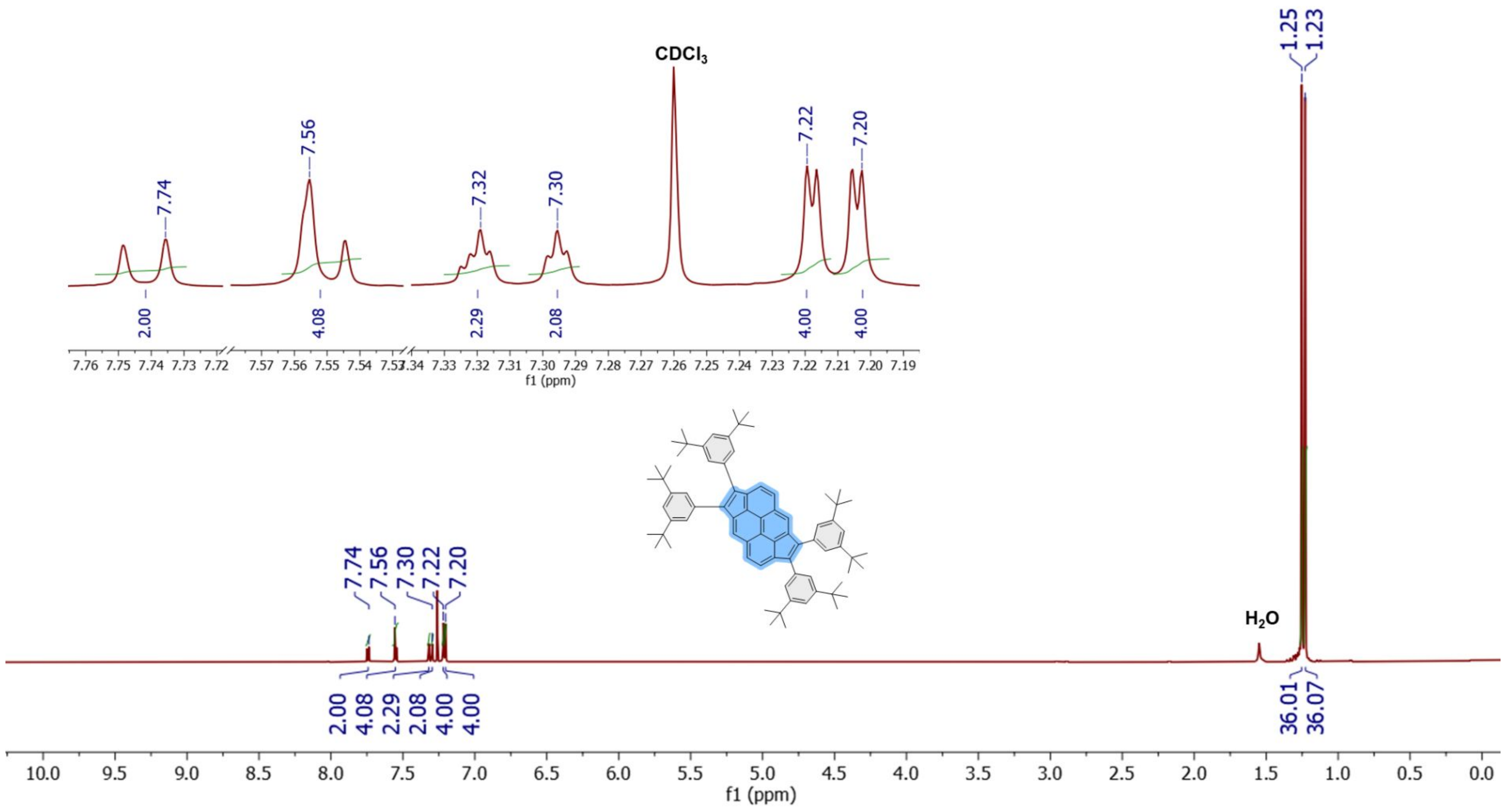

Figure S6: ^{1}H NMR spectrum of **DCP3** ($CDCl_3$, 600 MHz)

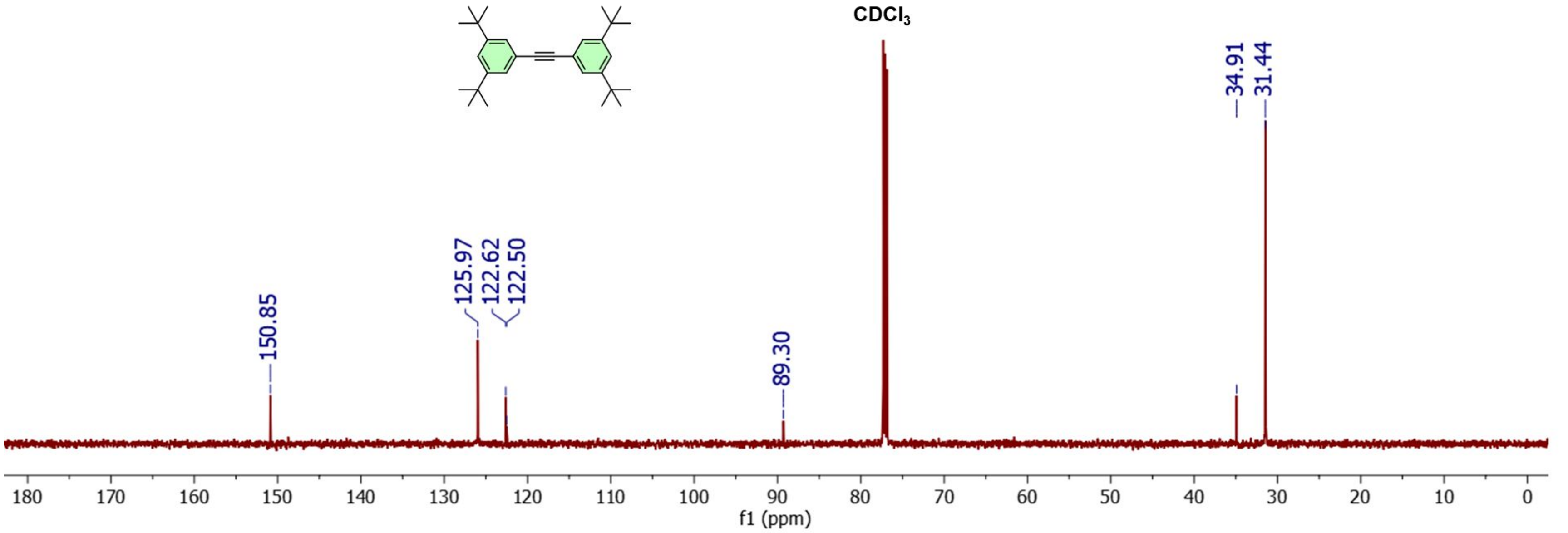

Figure S7: ^{13}C NMR spectrum of **2** ($CDCl_3$, 150 MHz)

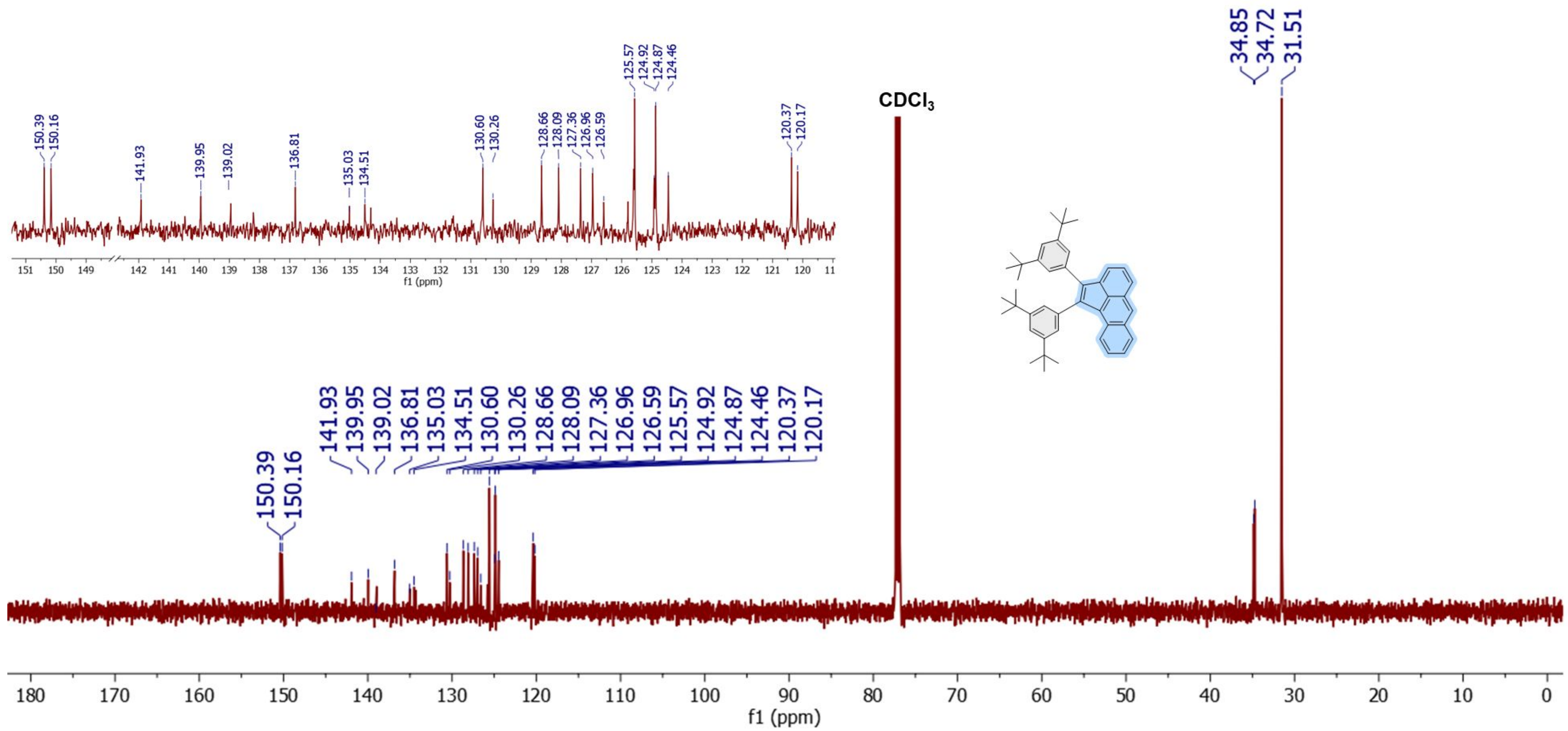

Figure S8: ^{13}C NMR spectrum of **MCP1** ($CDCl_3$, 150 MHz)

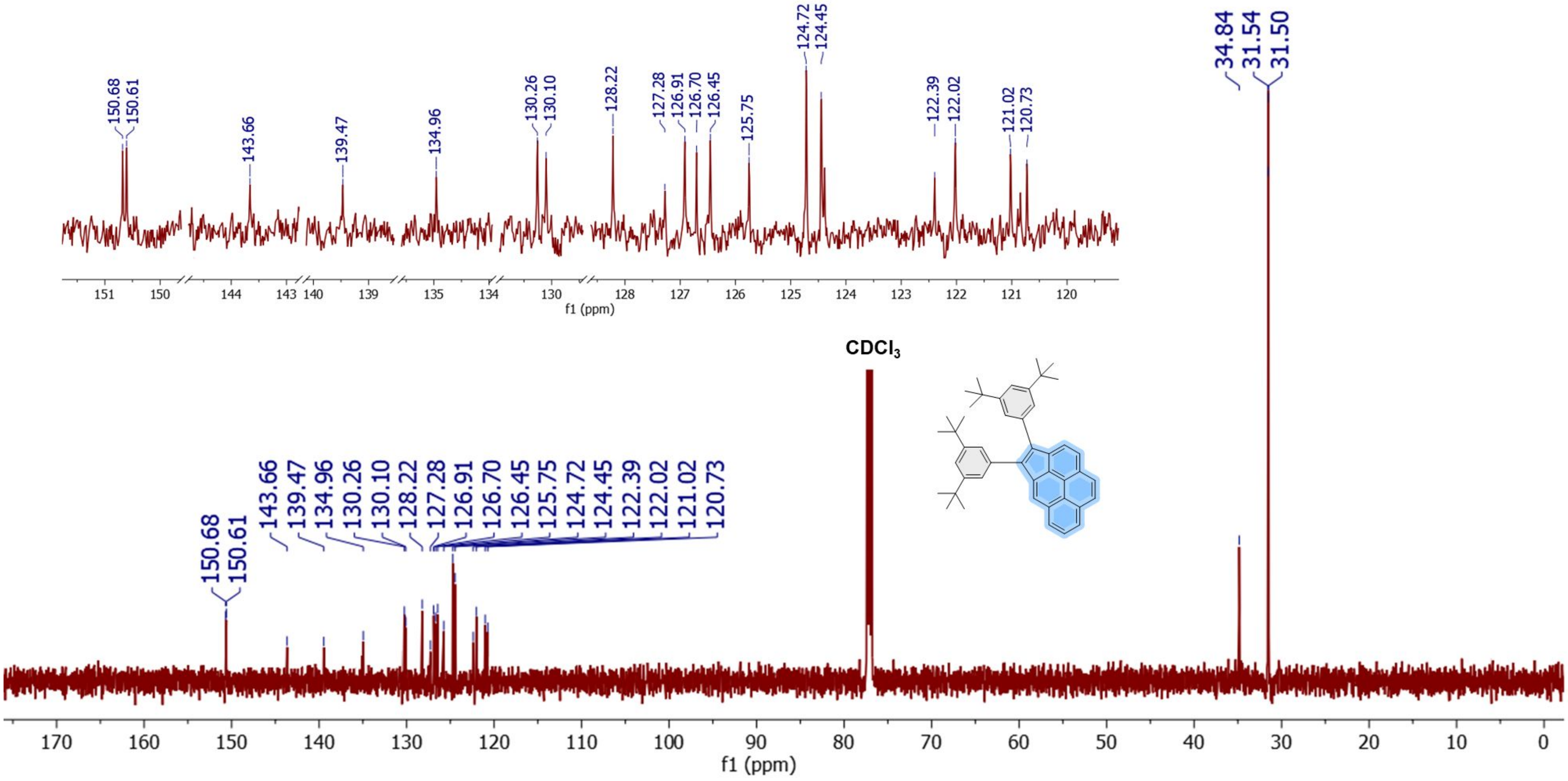

Figure S9: ^{13}C NMR spectrum of **MCP2** ($CDCl_3$, 150 MHz)

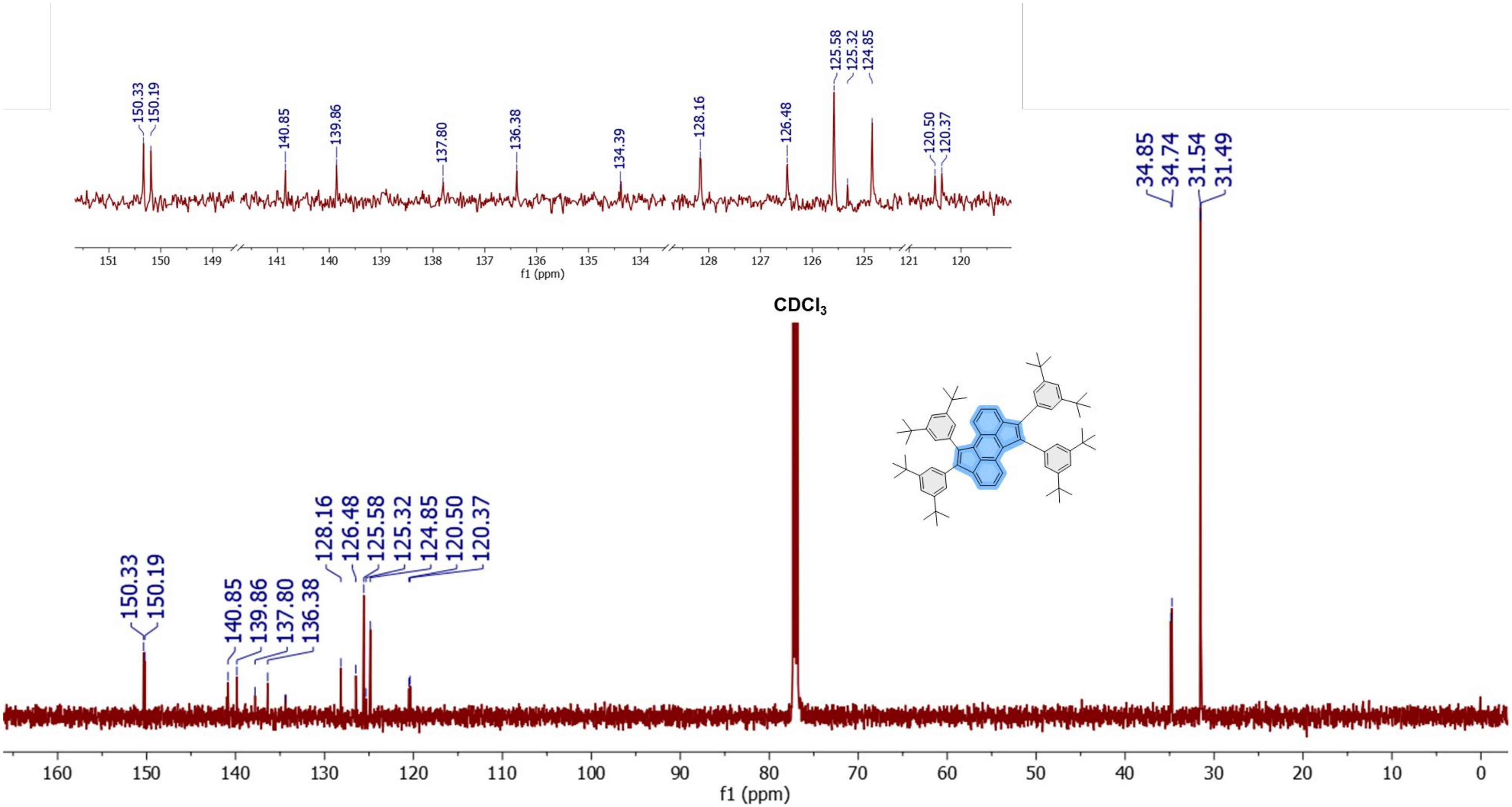

Figure S10: ^{13}C NMR spectrum of **DCP1** ($CDCl_3$, 150 MHz)

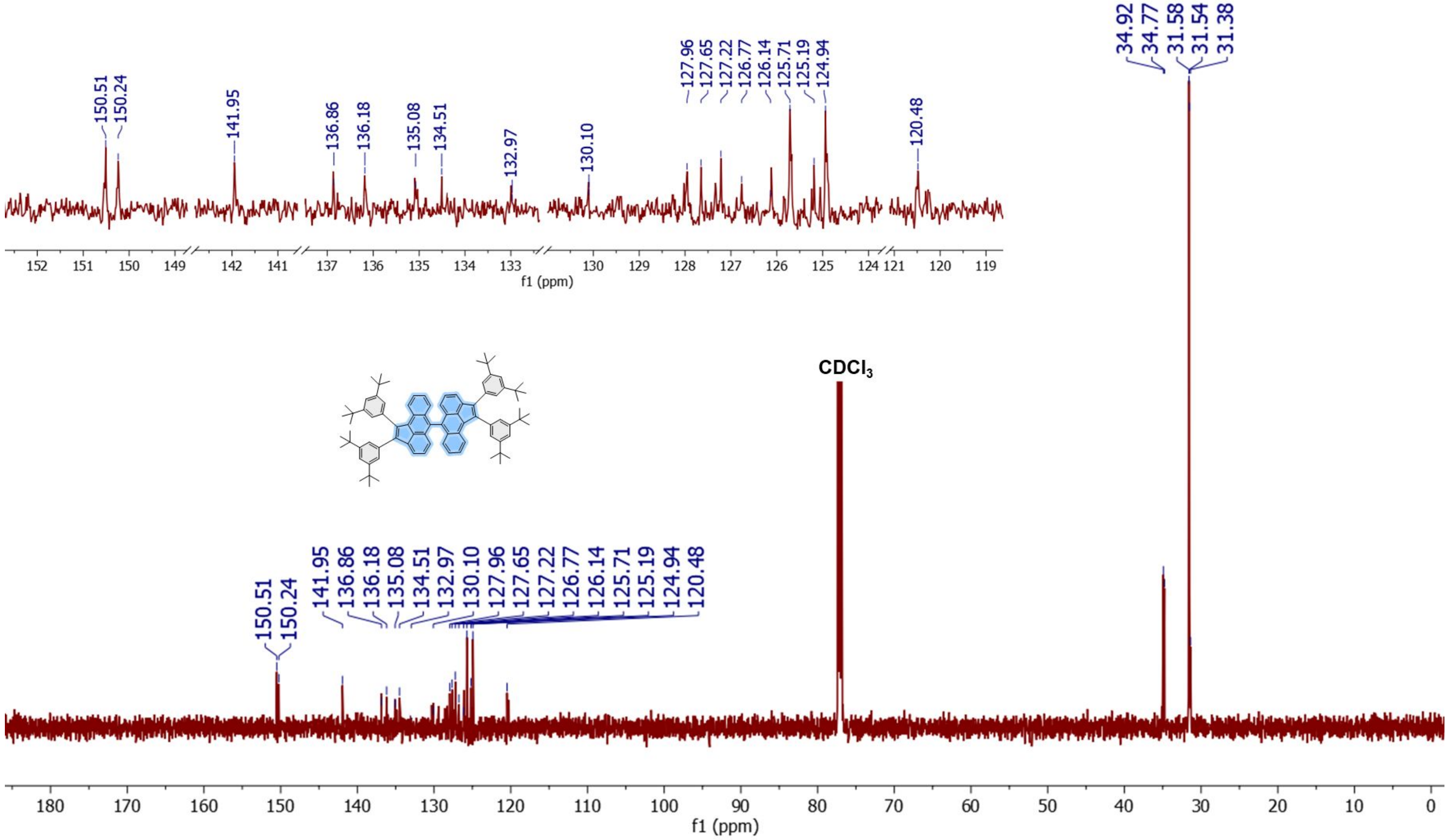

Figure S11: ^{13}C NMR spectrum of **DCP2** ($CDCl_3$, 150 MHz)

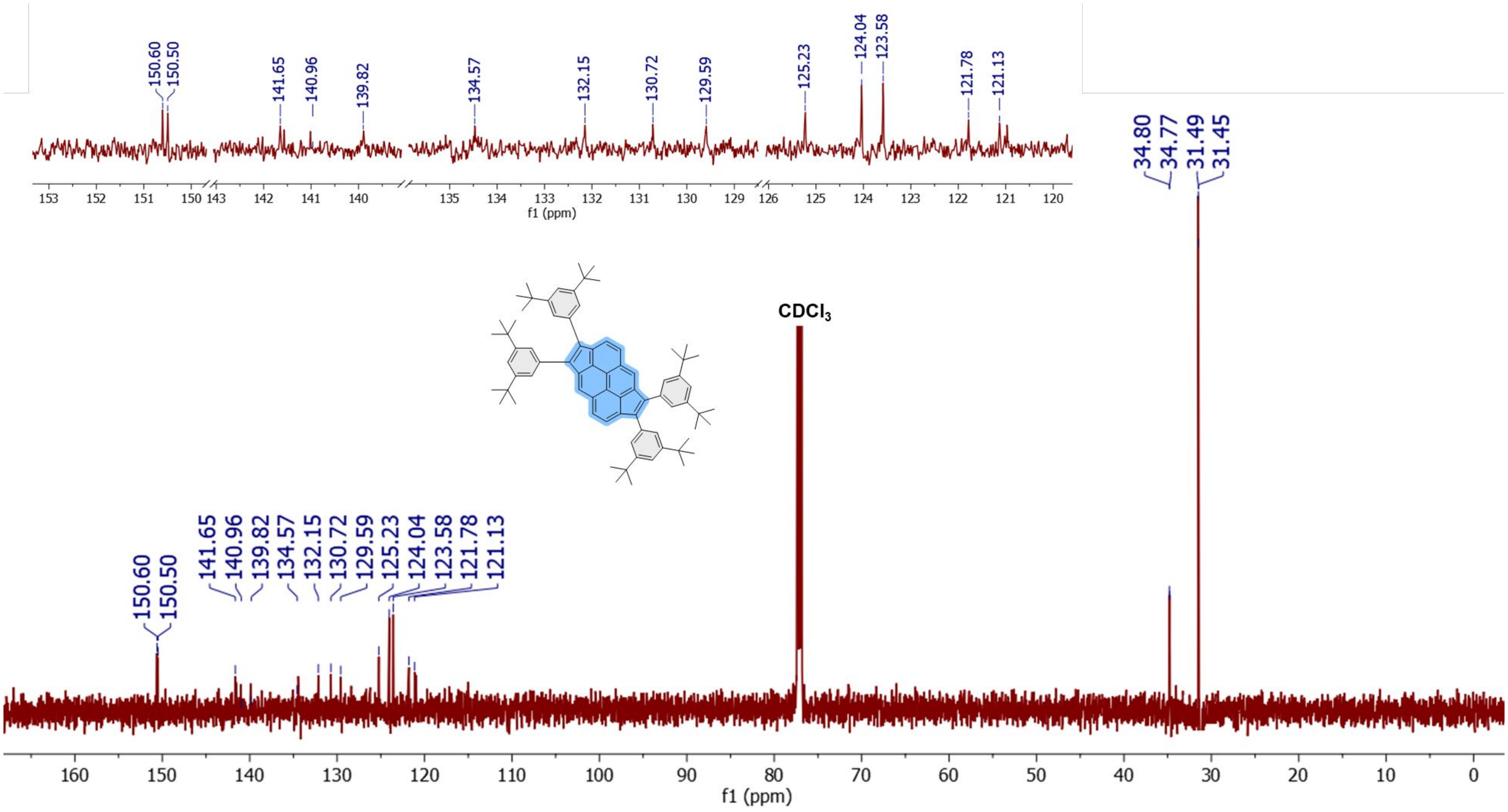

Figure S12: ^{13}C NMR spectrum of **DCP3** ($CDCl_3$, 150 MHz)

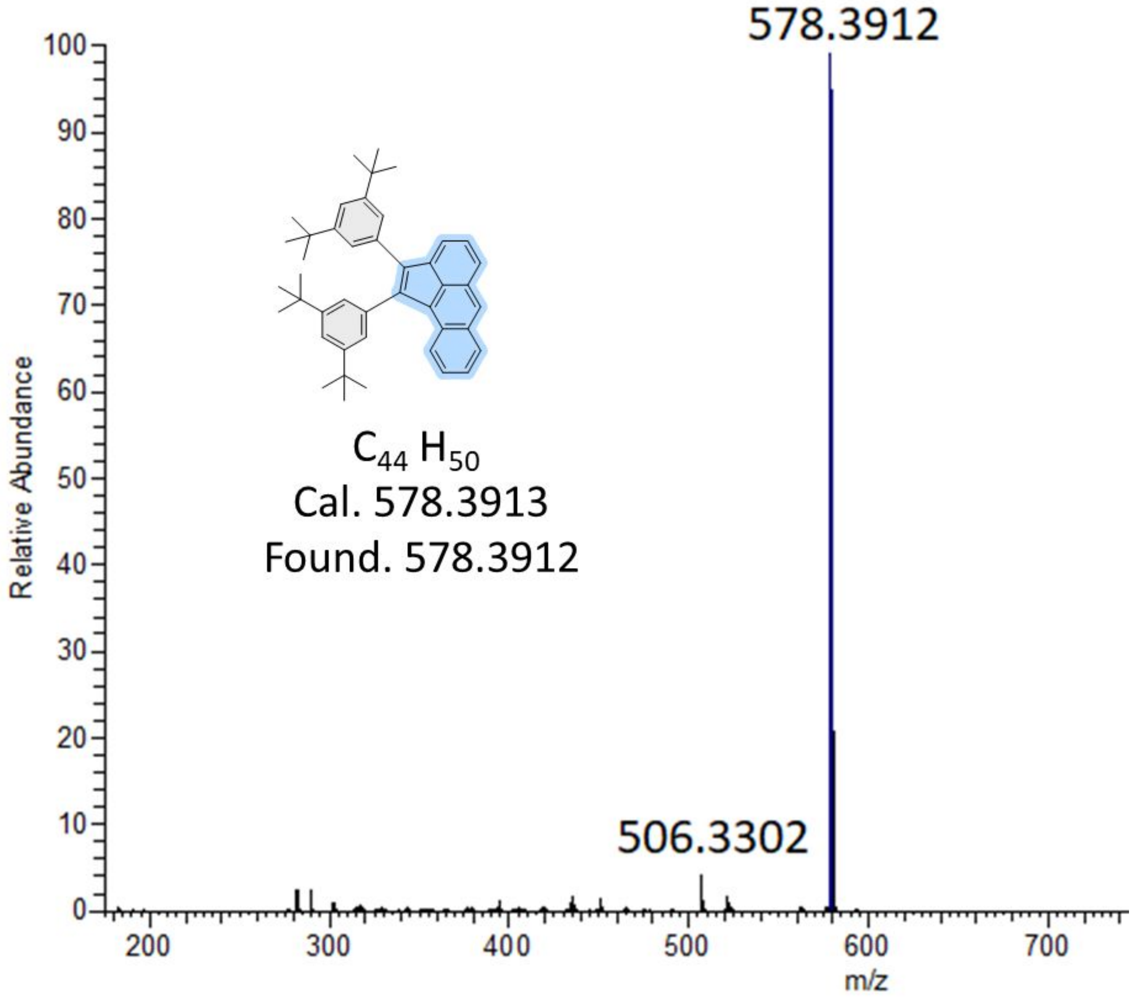

Figure S13: EI-HRMS spectra of **MCP1**

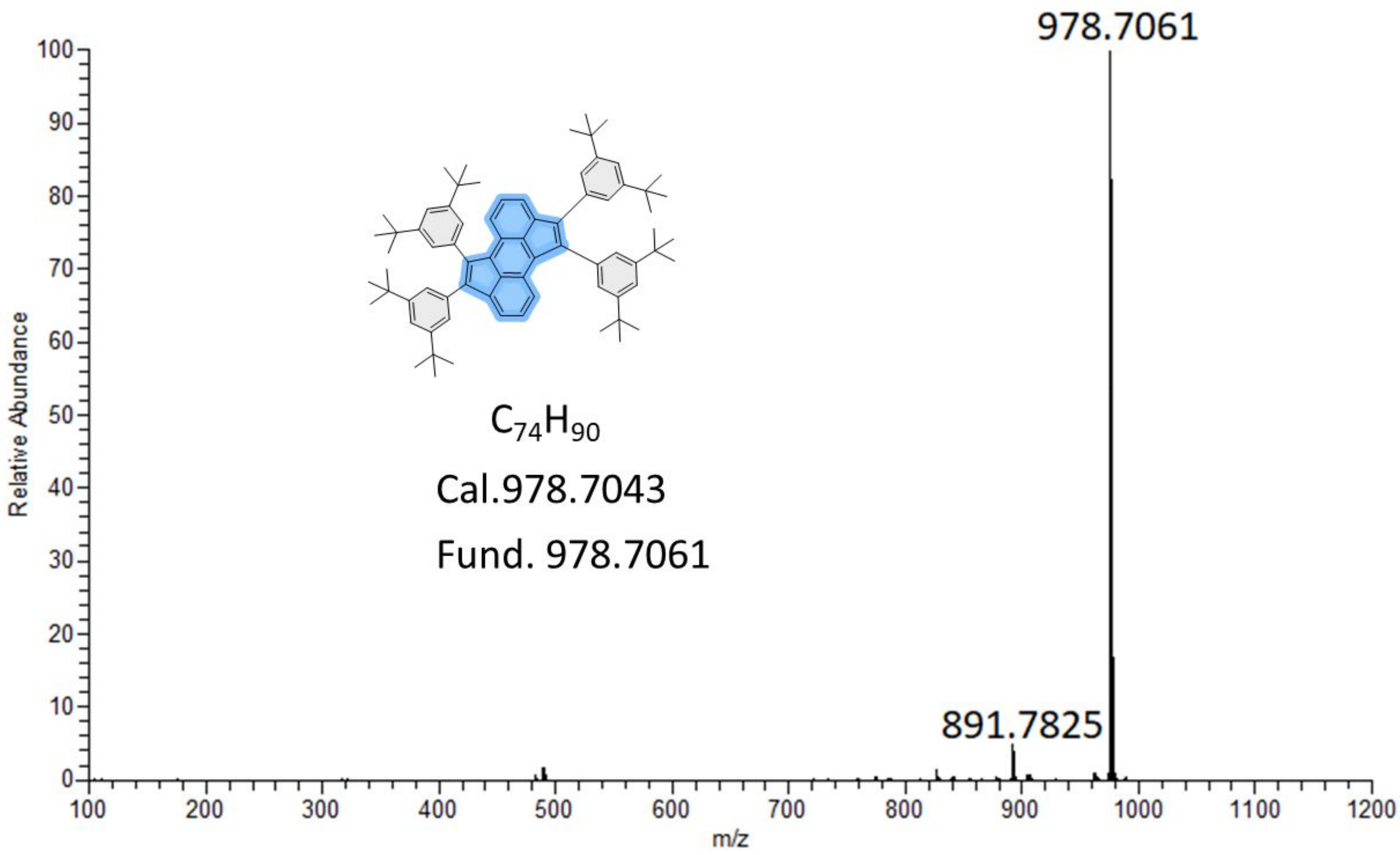

Figure S14: EI-HRMS spectra of **DCP1**

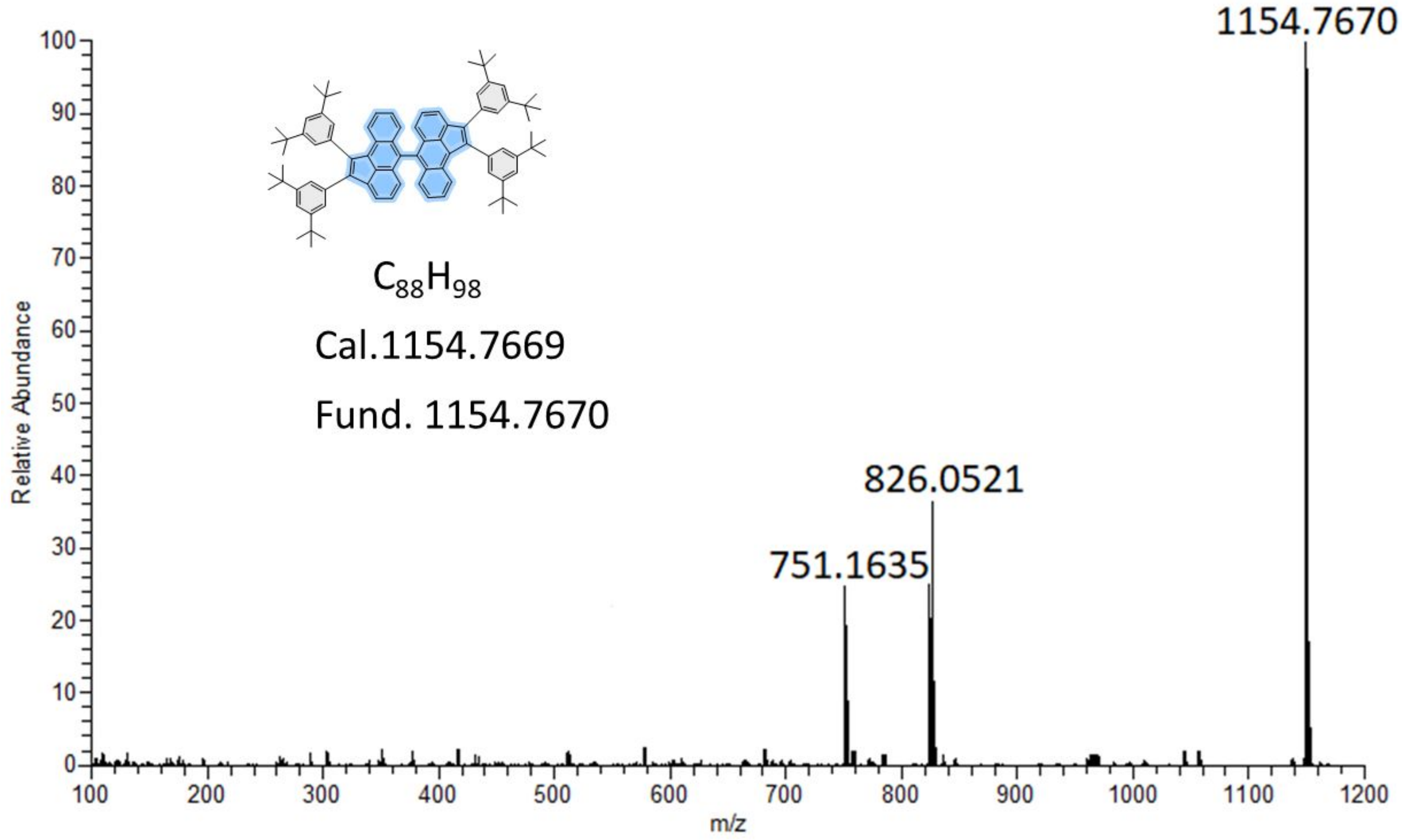

Figure S15: EI-HRMS spectra of **DCP2**

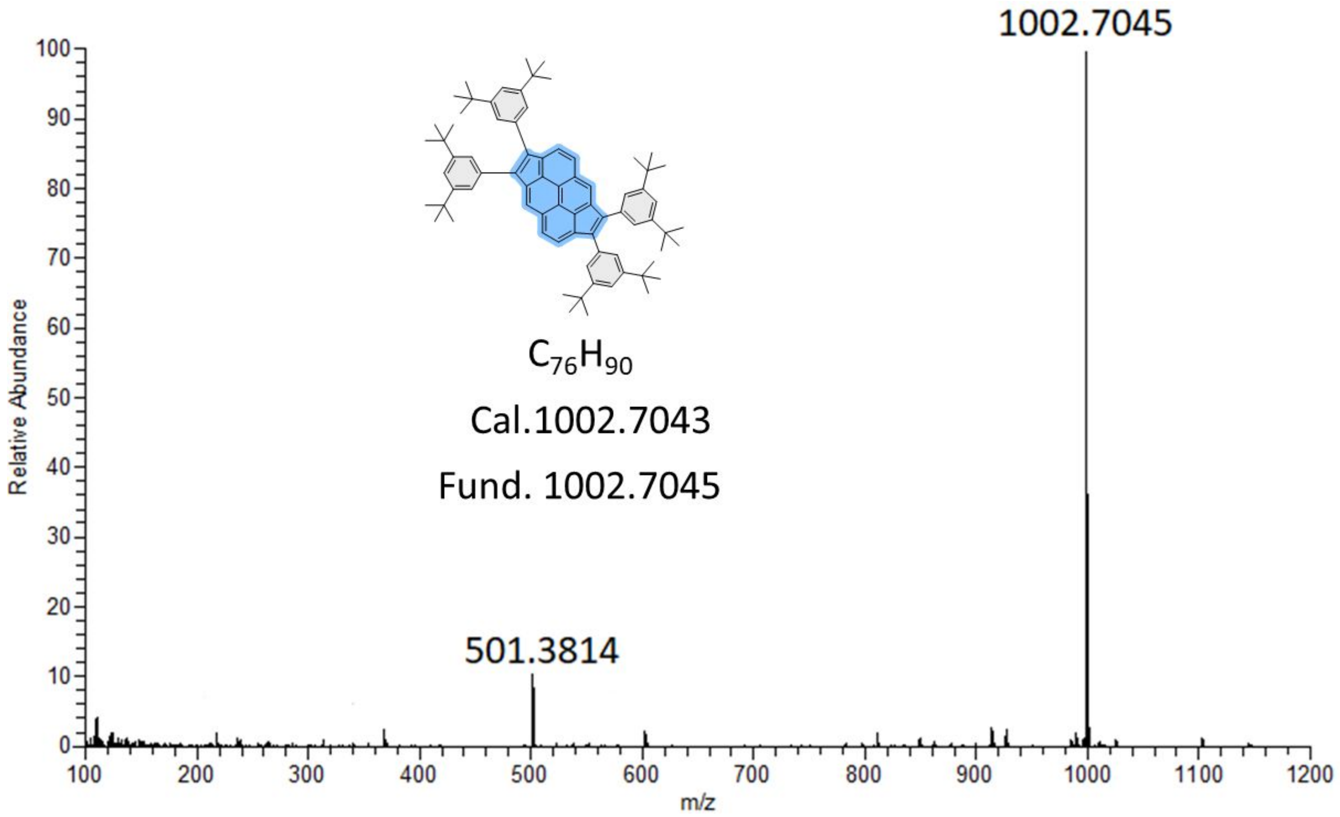

Figure S16: EI-HRMS spectra of **DCP3**

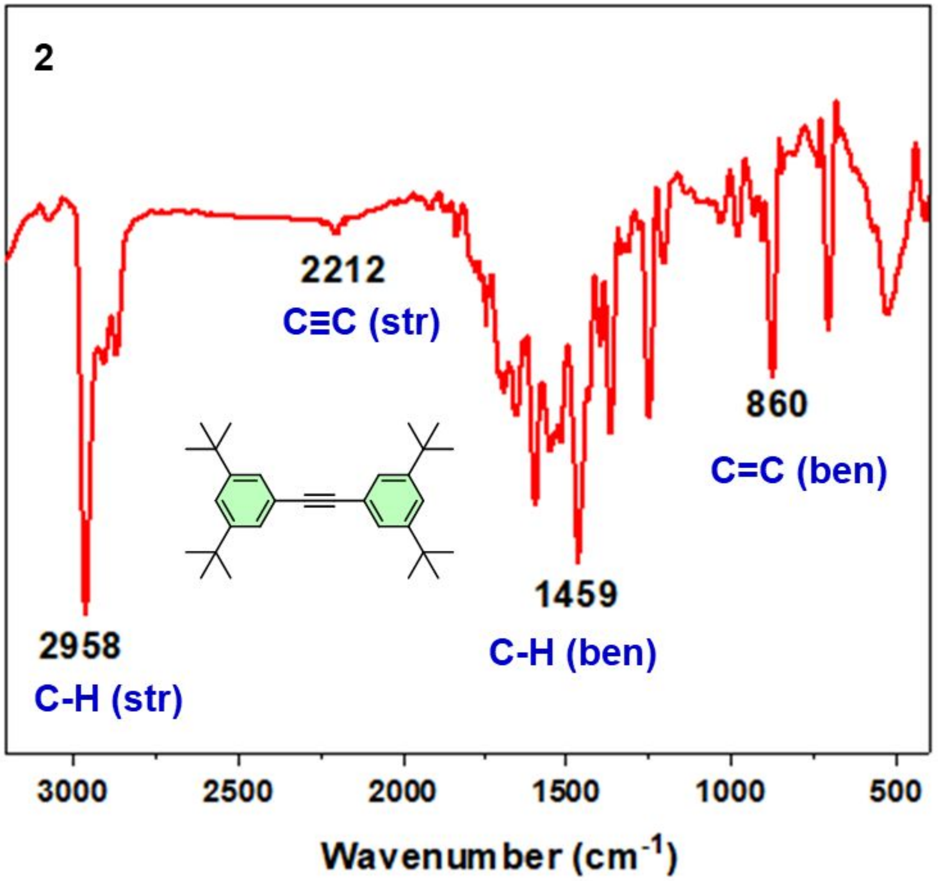

Figure S17: FT-IR spectra of **2**

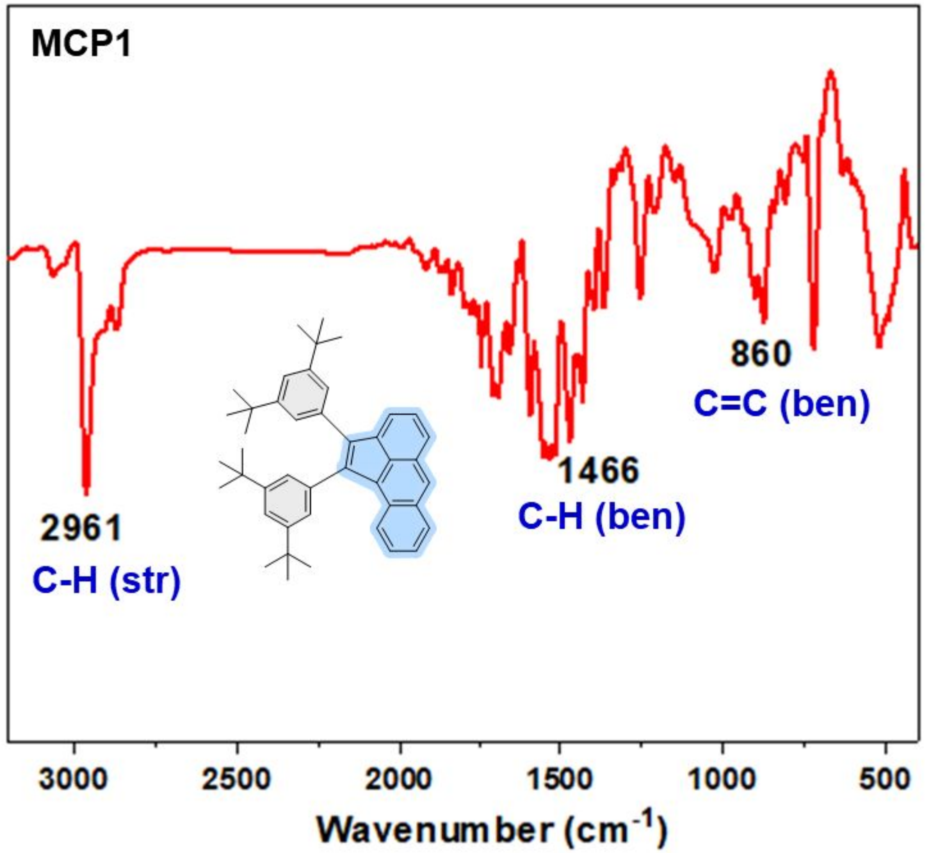

Figure S18: FT-IR spectra of **MCP1**

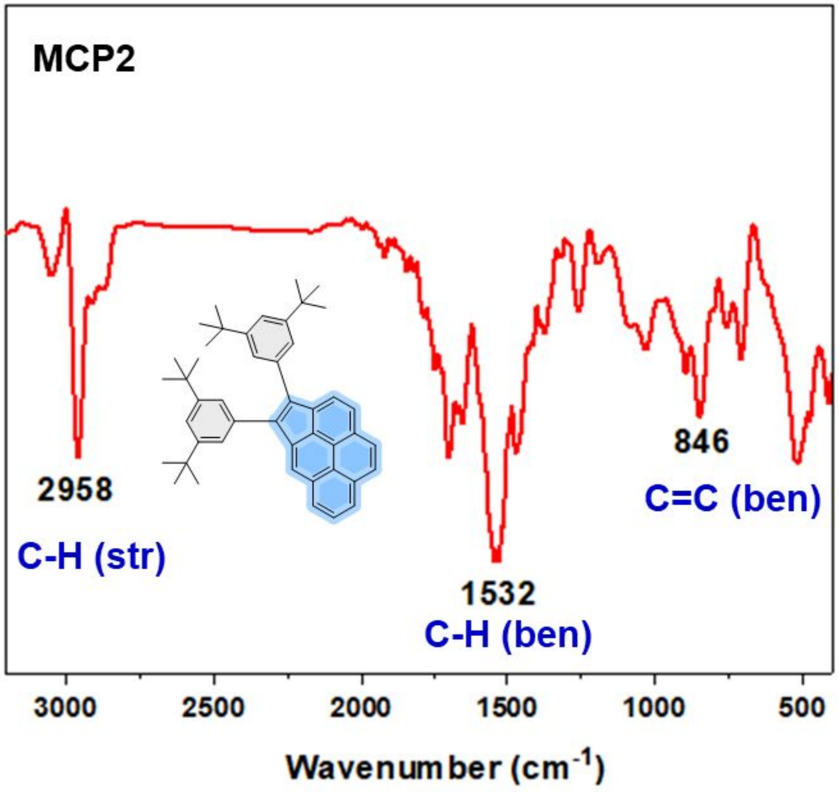

Figure S19: FT-IR spectra of **MCP2**

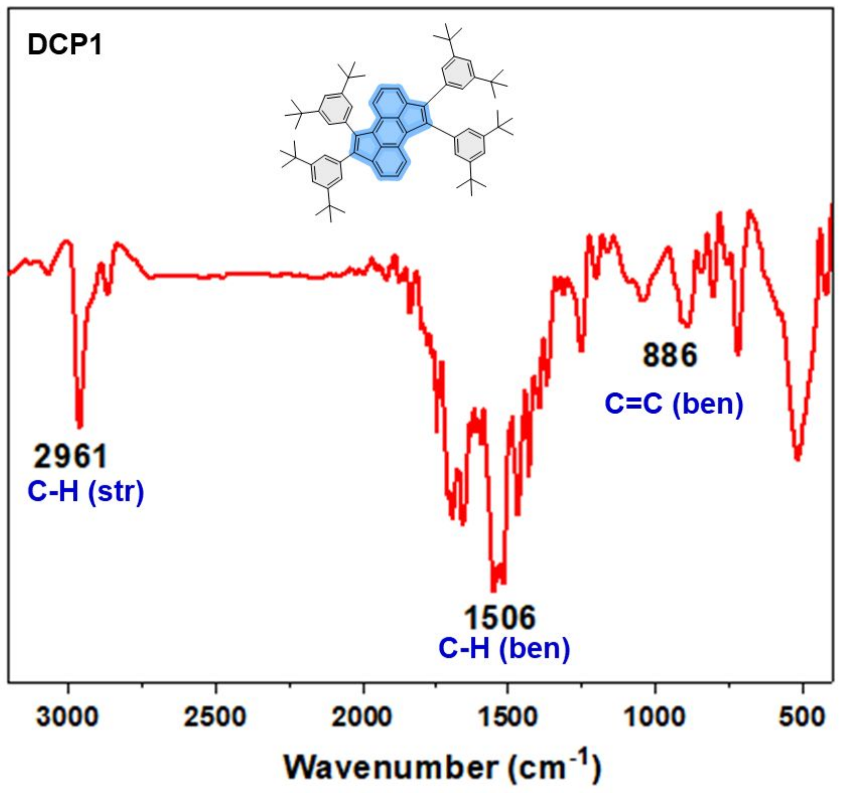

Figure S20: FT-IR spectra of **DCP1**

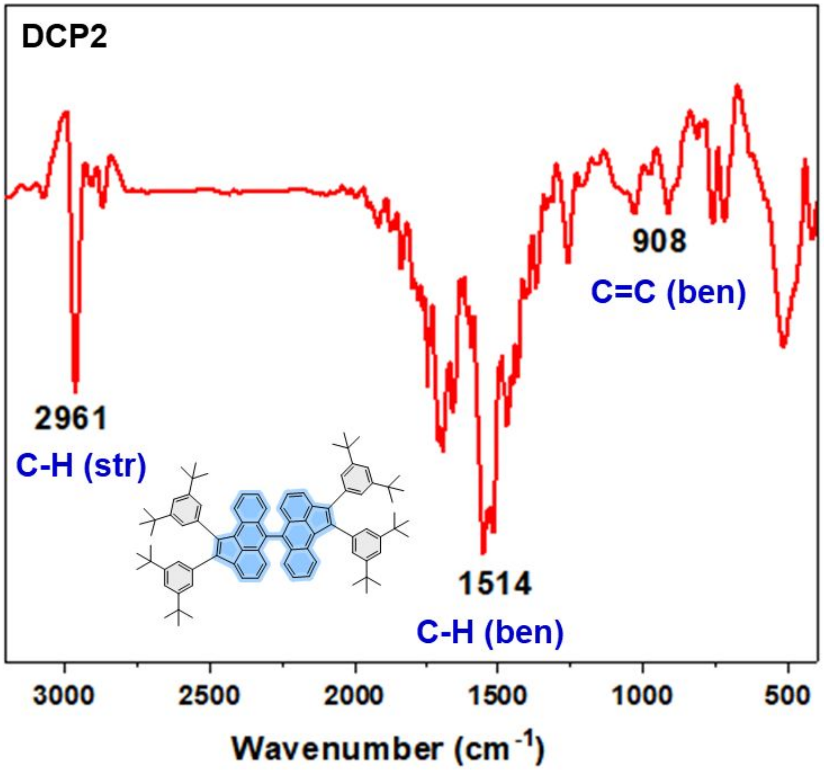

Figure S21: FT-IR spectra of **DCP2**

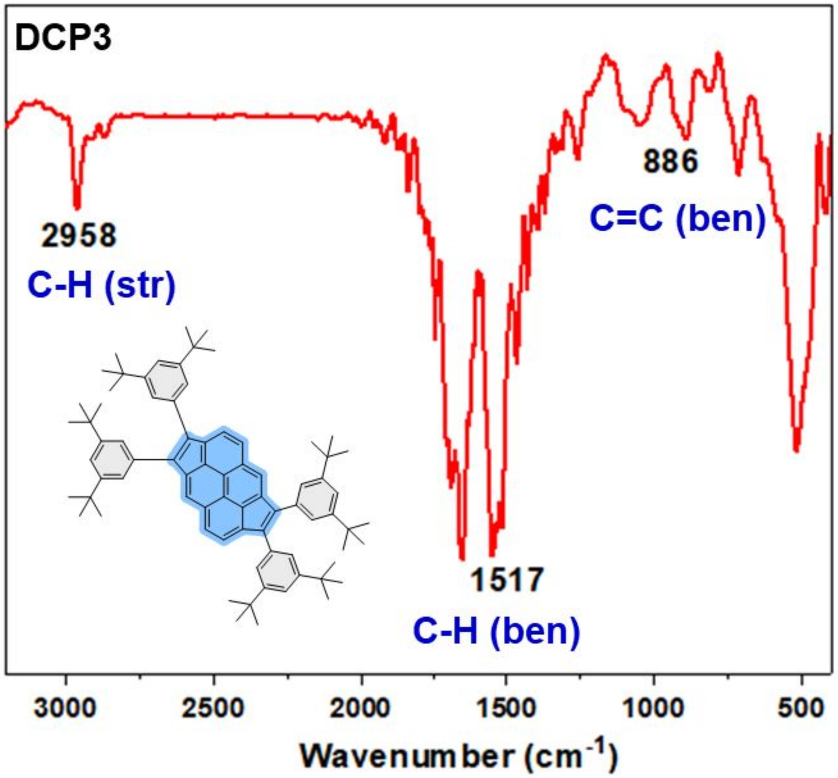

Figure S22: FT-IR spectra of **DCP3**

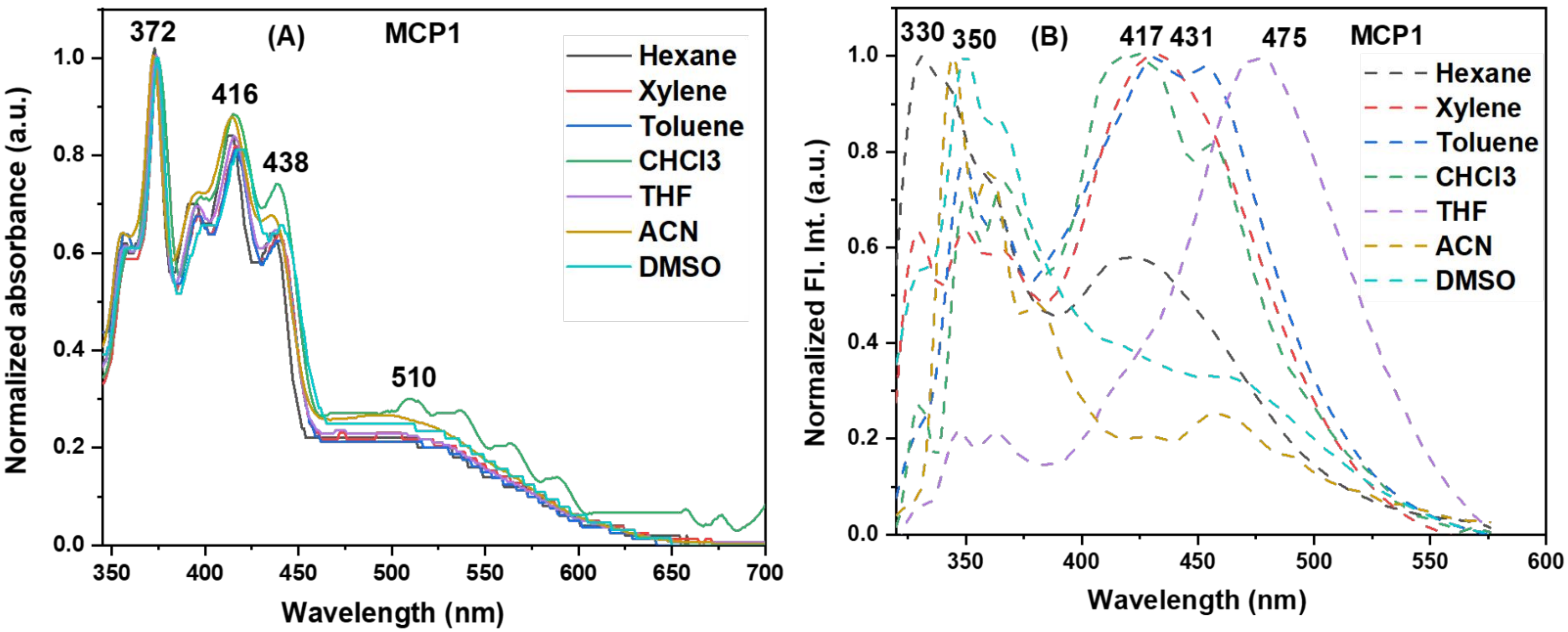

Figure S23: Normalized UV-Vis absorption (A) and emission spectra (B) of **MCP1** in different solvents (THF= tetrahydrofuran, ACN = Acetonitrile, DMSO = dimethyl sulfoxide, $C_M = 10^{-6}$ M (excitation wavelength = 372 nm).

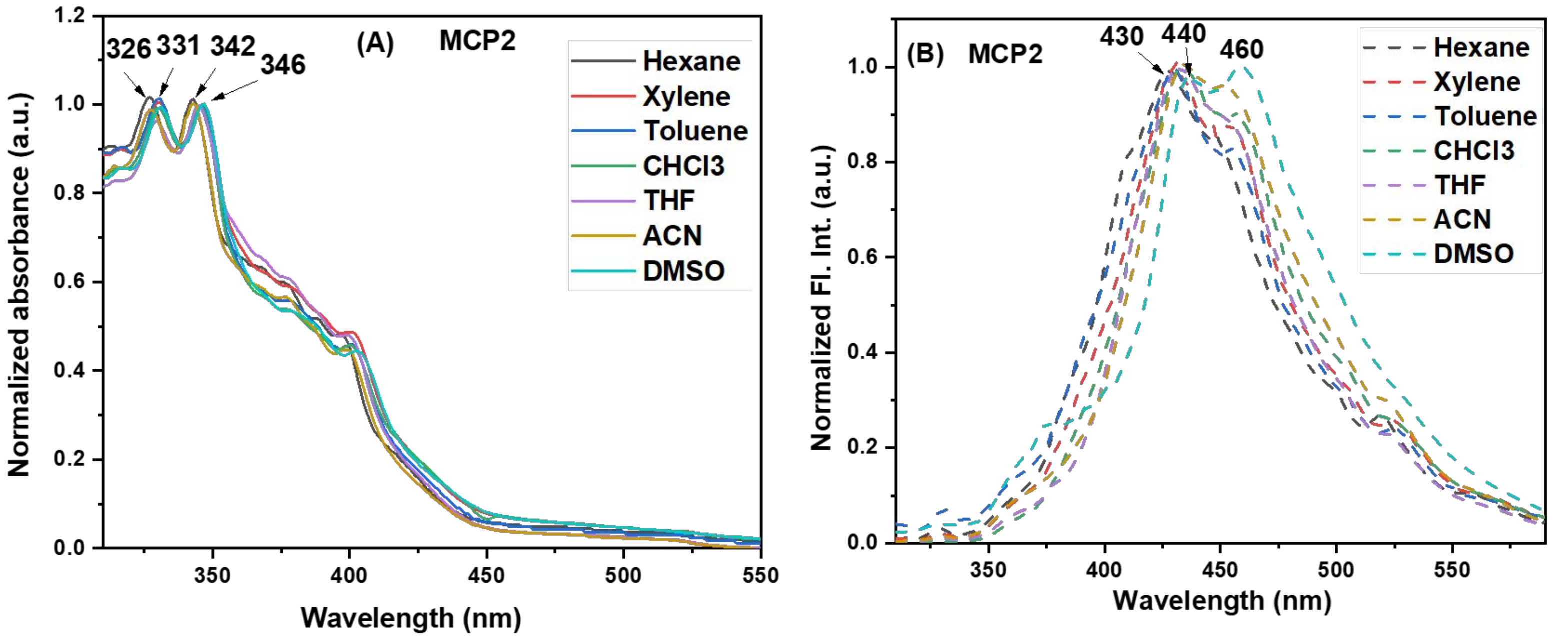

Figure S24: Normalized UV-Vis absorption (A) and emission spectra (B) of **MCP2** in different solvents (THF= tetrahydrofuran, ACN = Acetonitrile, DMSO = dimethyl sulfoxide, CM = 10^{-6} M (excitation wavelength = 326 nm).

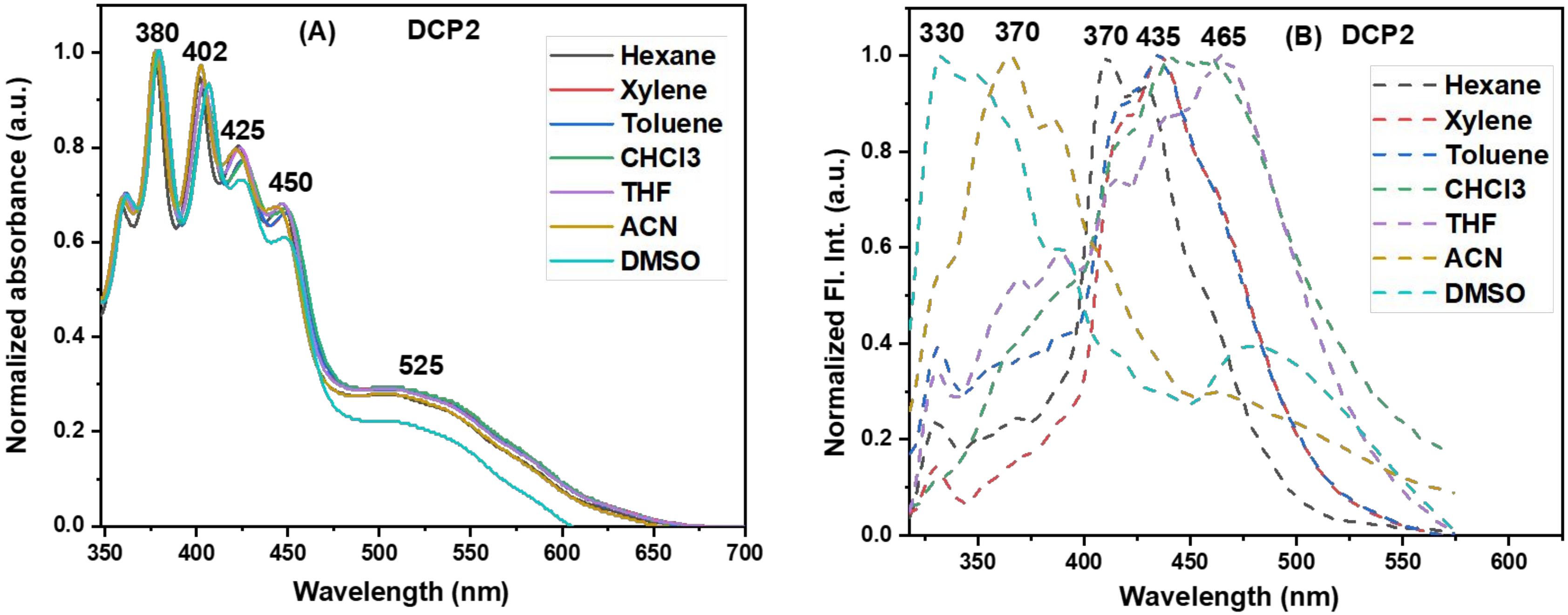

Figure S25: Normalized UV-Vis absorption (A) and emission spectra (B) of **DCP2** in different solvents (THF= tetrahydrofuran, ACN = Acetonitrile, DMSO = dimethyl sulfoxide, CM = 10^{-6} M (excitation wavelength = 380 nm).

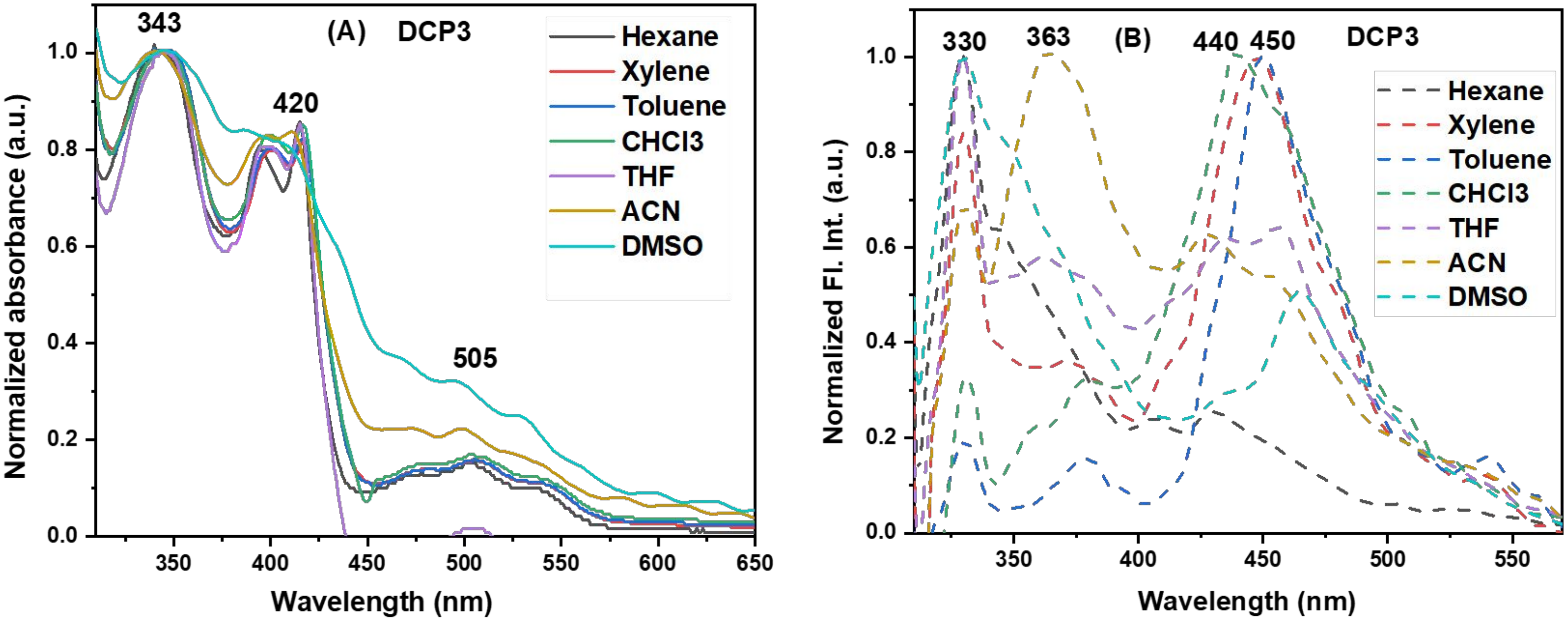

Figure S26: Normalized UV-Vis absorption (A) and emission spectra (B) of **DCP3** in different solvents (THF= tetrahydrofuran, ACN = Acetonitrile, DMSO = dimethyl sulfoxide, CM = 10^{-6} M (excitation wavelength = 343 nm).

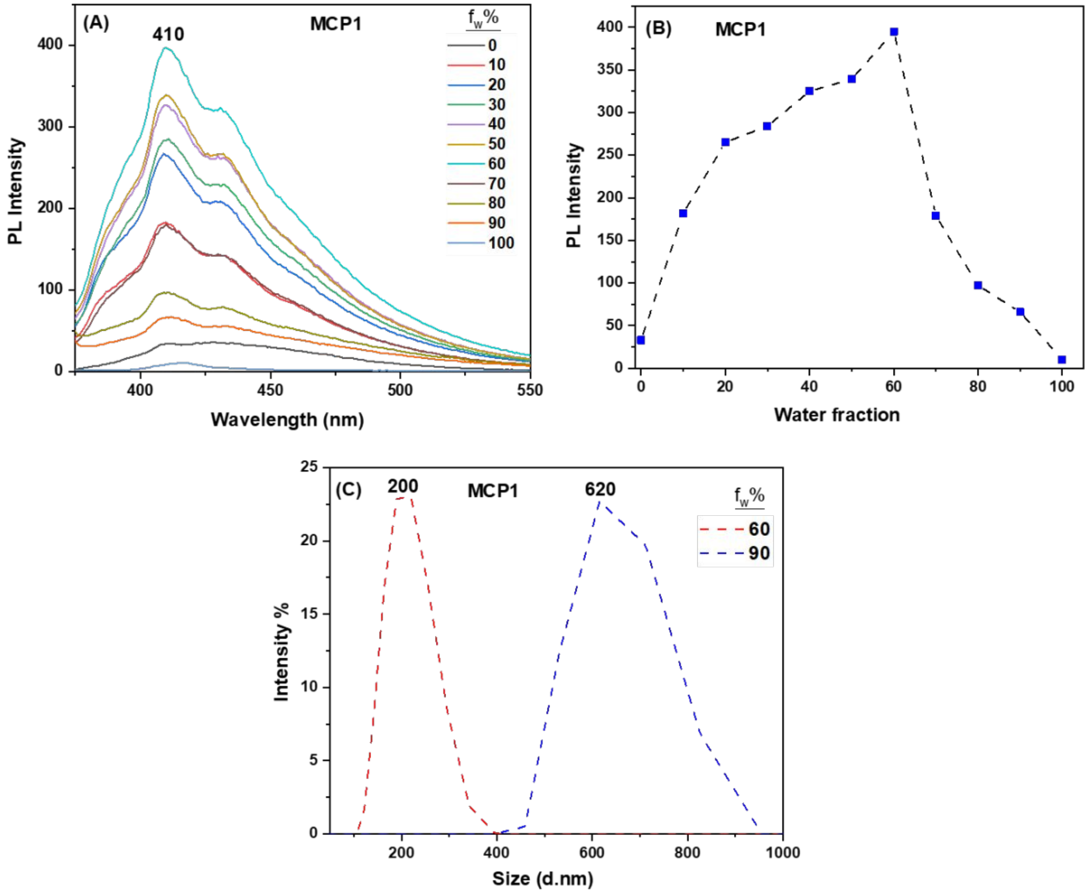

Figure S27: (A) Emission spectra of **MCP1** in THF/water mixtures (0–100 %) (B) Plot of maximum emission intensity of **MCP1** versus water fraction (C) Dynamic light-scattering (DLS) spectra of **MCP1** in THF/water, f_w of 60% (red dashed line) and 90% (blue dashed line)

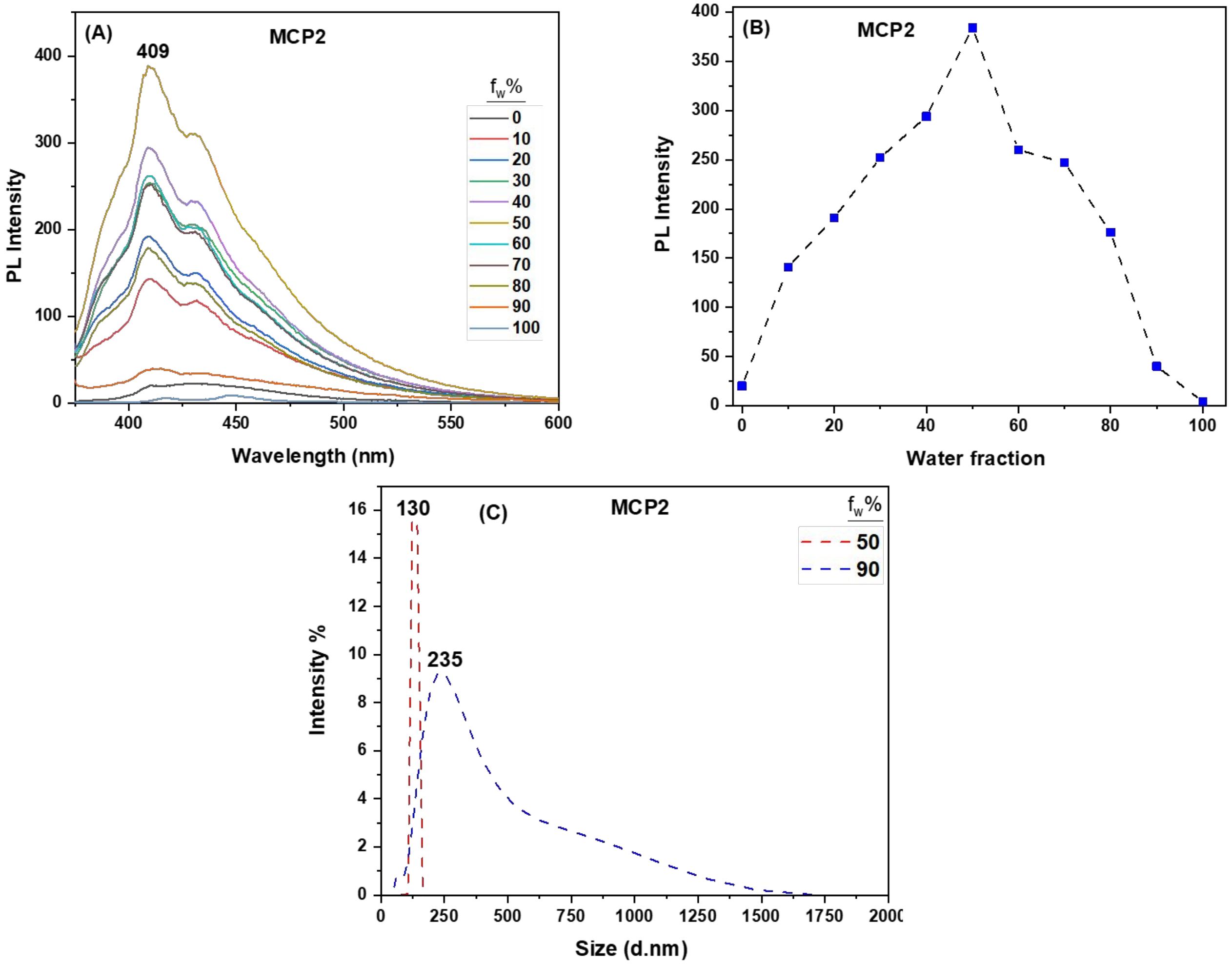

Figure S28: (A) Emission spectra of **MCP2** in THF/water mixtures (0–100 %) (B) Plot of maximum emission intensity of **MCP2** versus water fraction (C) Dynamic light-scattering (DLS) spectra of **MCP2** in THF/water, f_w of 50% (red dashed line) and 90% (blue dashed line)

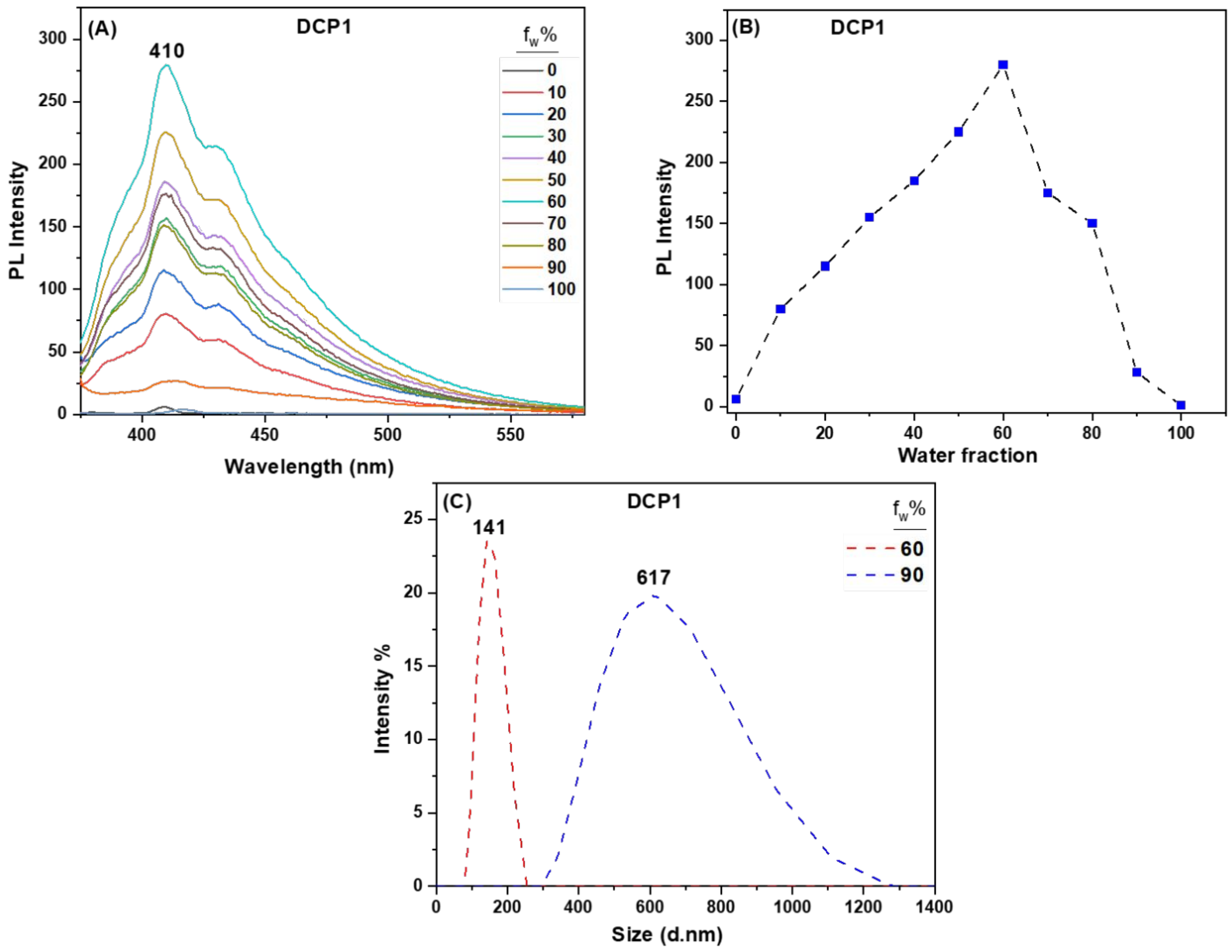

Figure S29: (A) Emission spectra of **DCP1** in THF/water mixtures (0–100 %) (B) Plot of maximum emission intensity of **DCP1** versus water fraction (C) Dynamic light-scattering (DLS) spectra of **DCP1** in THF/water, f_w of 60% (red dashed line) and 90% (blue dashed line)

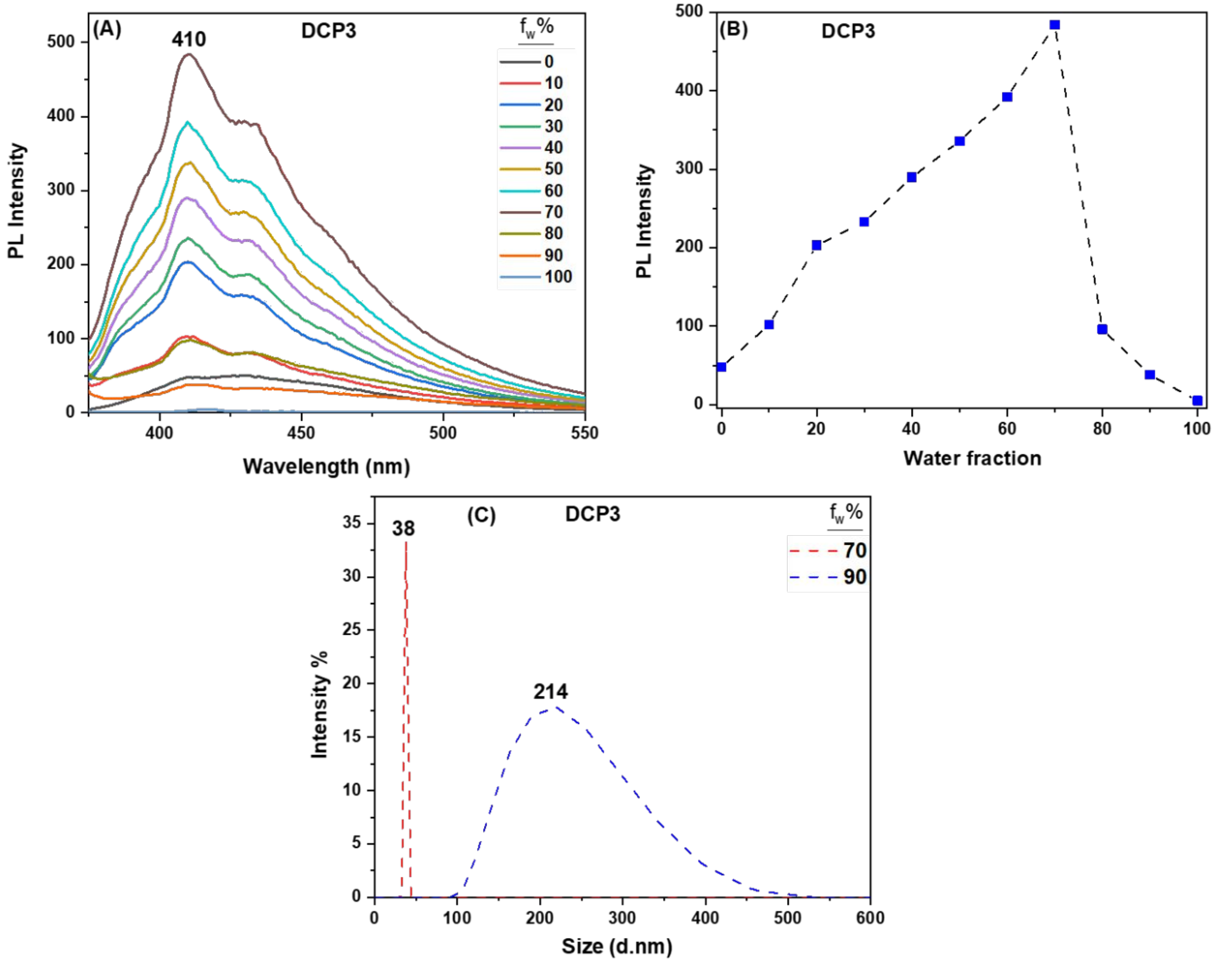

Figure S30: (A) Emission spectra of **DCP3** in THF/water mixtures (0–100 %) (B) Plot of maximum emission intensity of **DCP3** versus water fraction (C) Dynamic light-scattering (DLS) spectra of **DCP3** in THF/water, f_w of 70% (red dashed line) and 90% (blue dashed line)

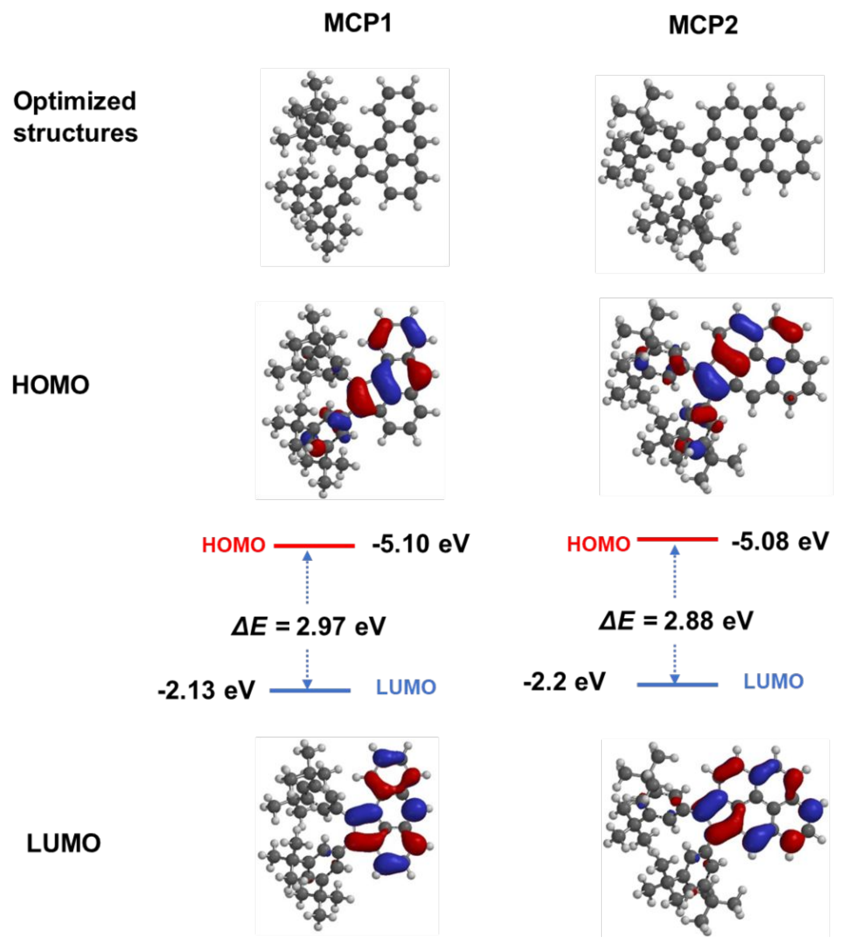

Figure S31: Optimized structures and molecular orbital amplitude plots of HOMO and LUMO energy levels of **MCP1** and **MCP2** calculated using the B3LYP/6-31G* basis set.

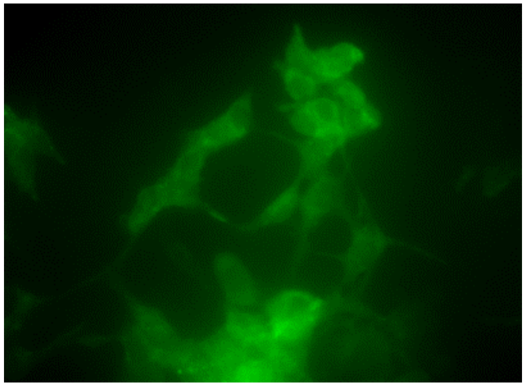

Figure S32: Optical microscopy images showing uptake of AIE dots (**DCP2**) emission of RAW cells under fluorescence microscope.

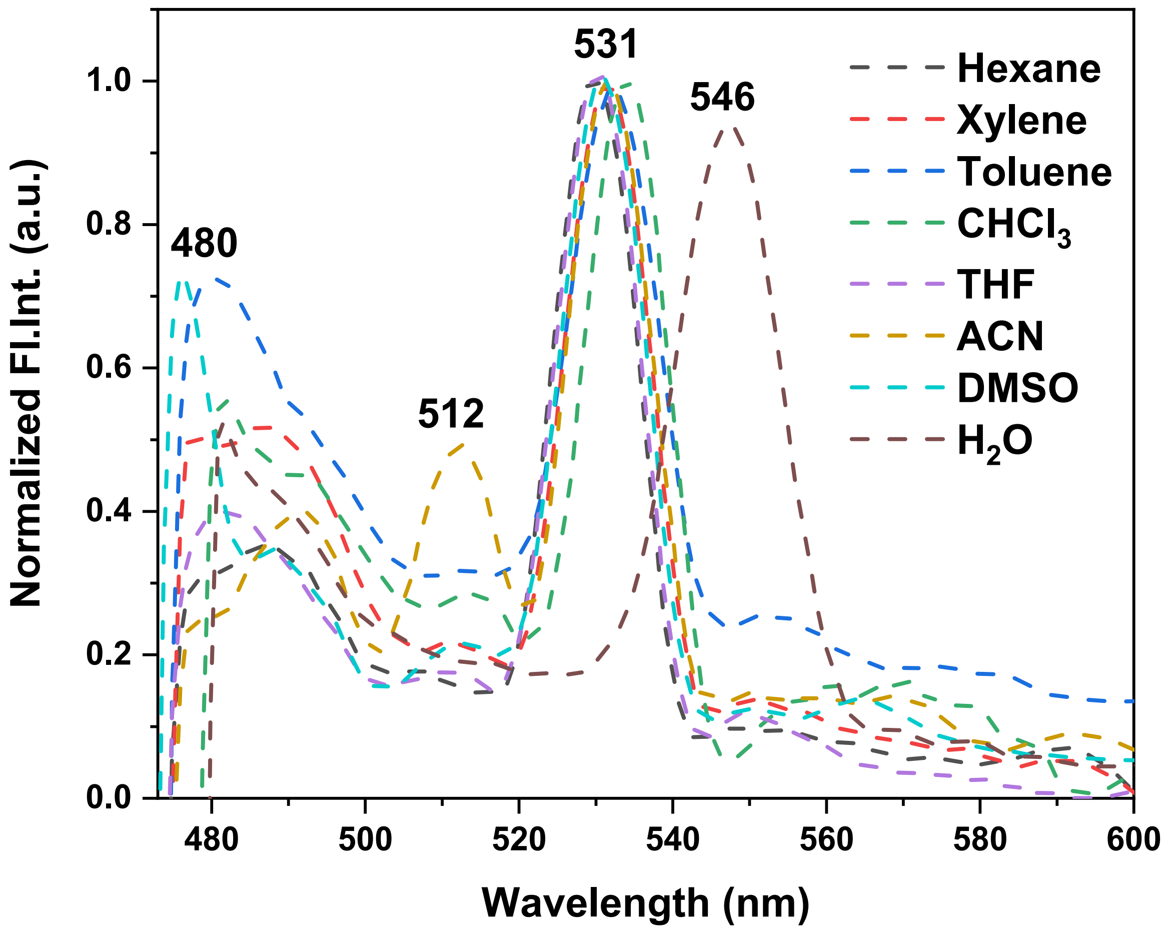

Figure S33: Emission spectra of **DCP2** in different solvents (THF= tetrahydrofuran, ACN = Acetonitrile, DMSO = dimethyl sulfoxide, CM = 10^{-6} M (excitation wavelength = 458 nm).
